# Functional and structural insights into a thermostable (*S*)-selective amine transaminase and its improved substrate scope by protein engineering

**DOI:** 10.1007/s00253-025-13536-9

**Published:** 2025-08-12

**Authors:** Stefania Patti, Simone A. De Rose, Michail N. Isupov, Ilaria Magrini Alunno, Sergio Riva, Erica Elisa Ferrandi, Jennifer A. Littlechild, Daniela Monti

**Affiliations:** 1 Istituto Di Scienze E Tecnologie Chimiche “G. Natta” (SCITEC), CNR, Milan, Italy; 2 Department of Pharmaceutical Sciences, University of Milan, Milan, Italy; 3 Henry Wellcome Building for Biocatalysis, Biosciences, Faculty of Health and Life Sciences, University of Exeter, Exeter, UK

**Keywords:** Biocatalysis, Amine transaminases, Substrate specificity, Stereoselectivity, Protein engineering, Crystallographic structure

## Abstract

**Abstract:**

A (*S*)-selective amine transaminase from a *Streptomyces* strain, Sbv333-ATA, is a biocatalyst showing both high thermostability with a melting temperature of 85 °C and broad substrate specificity for the amino acceptor. This enzyme was further characterized both biochemically and structurally. The Sbv333-ATA is stable in the presence of up to 20% (*v*/*v*) of the water-miscible cosolvents methanol, ethanol, acetonitrile, and dimethyl sulfoxide, and in biphasic systems with petroleum ether, toluene, and ethyl acetate as an organic phase. The enzyme showed also a good activity toward different amino donors, such as (*S*)-methylbenzylamine and 2-phenylethylamine, aliphatic mono- and di-amines, like propylamine and cadaverine, and selected amino acids. However, more sterically hindered aromatic amines were not accepted. Based on the knowledge of the three-dimensional structures obtained, a rational approach to site specific mutagenesis was carried out to broaden the substrate specificity of Sbv333-ATA. The mutant W89A showed the highest activity toward bulky amines as substrates, such as the diaromatic compound 1,2-diphenylethylamine. The 3D structures of the holo and inhibitor gabaculine bound forms of native Sbv333-ATA, and holo W89A and F61C mutants were determined at high resolutions of 1.49, 1.24, and 1.31 (both mutants) Å, respectively. These structures were important for revealing further details of the active site binding pockets of the Sbv333-ATA and its mechanism.

**Key points:**

• *Sbv333-ATA is a highly thermostable transaminase with a broad substrate scope.*

• *Sbv333-ATA remains active in various organic cosolvents and biphasic systems.*

• *Mutant W89A expands substrate range to accept bulky diaromatic amines.*

**Graphical abstract:**

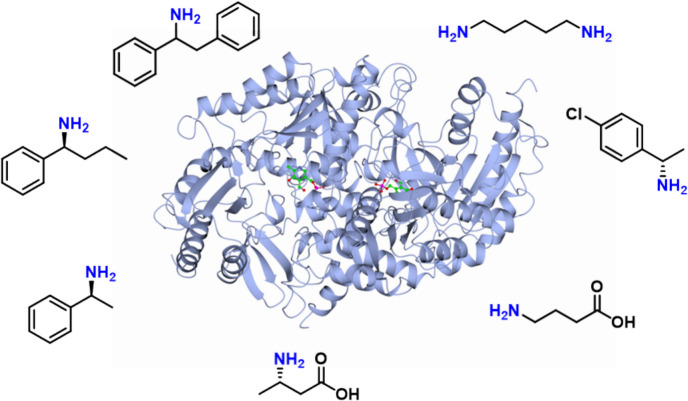

**Supplementary Information:**

The online version contains supplementary material available at 10.1007/s00253-025-13536-9.

## Introduction

Transaminases (E.C. 2.6.1.x) are pyridoxal-5′-phosphate (PLP)-dependent enzymes that catalyze the synthesis of primary chiral amines by transferring an amino group from an amine donor to a prochiral ketone (acceptor) (Hughes 2018). In particular, amine transaminases (ATAs), belonging to class III ω-transaminases (Mehta et al. 1993), accept a broad spectrum of amine donors and acceptors in stereoselective reductive amination processes and are valuable industrial tools for the synthesis of enantiomerically pure chiral amines (Kelly et al. 2018; Ferrandi and Monti 2018). Their significance is particularly evident in sectors such as the pharmaceutical industry, where stringent requirements for the enantiopurity of molecules demand sophisticated and precise synthetic methodologies (Ghislieri and Turner 2014; Fuchs et al. 2015). Furthermore, from an industrial perspective, ATAs represent an interesting alternative to conventional chemical synthesis methods by eliminating the necessity for heavy metal catalysts, and they operate under ambient temperature and physiological pH (Constable et al. 2007). This biocatalytic approach represents a sustainable method for chemical synthesis (Alcántara et al. 2022).

The catalytic mechanism of ATAs involves a two-step process: firstly, the deamination of an amine donor, leading to the release of the amine donor product, and secondly, the amination of an amine acceptor (keto acid, ketone, or aldehyde), resulting in the production of a new amino acid or amine. The pyridoxal phosphate (PLP) cofactor, bound to a catalytic lysine of the enzyme through an imine bond, assists the reaction by serving as a transient molecular shuttle for the amino group. By the end of the reaction, the cofactor liberates the amino group and reverts to its original state (automatic cofactor recycling). This feature is a significant advantage as it eliminates the need for cofactor regeneration, unlike many other enzymes (Guo and Berglund 2017).

The determination of the three-dimensional structure of transaminases is of great significance in the fields of enzyme engineering and biocatalysis. To date, many crystal structures of different classes of transaminases with either (*R*) or (*S*) selectivity are available (Sayer et al. 2012; Midelfort et al. 2013; Łyskowski et al. 2014; Isupov et al. 2019). These studies provide detailed insights into the proteins’ structure, including the arrangement of key amino acids forming the enzyme active site. These insights are valuable for the development of engineered variants with specific features of stability, substrate scope, or enantiomeric selectivity, for the development of sustainable industrial processes and the synthesis of pharmaceutical compounds (Buller et al. 2023).

Wild-type ATAs usually exist as homodimers, or larger arrangements of homodimers such as tetramers and hexamers (Sayer et al. 2013; Isupov et al. 2019; Meng et al. 2022), with the active site located at the dimer interface and composed of amino acid residues from different subunits. In general, the catalytic site of ATAs can be divided into two distinct regions, specifically a large binding pocket (L pocket) and a small binding pocket (S pocket) (Genz et al. 2015). The existence of these two binding pockets imposes a constraint on substrate scope, as bulky substrates of industrial and pharmaceutical relevance are often not accepted by wild-type transaminases. This limitation has motivated researchers to undertake an enzyme engineering approach aimed at enlarging the size of the binding pockets, thereby expanding the substrate scope (Bornscheuer et al. 2012; Meng et al. 2022).

To date, several instances of engineered variants with broader substrate specificity have been reported in the literature (Han et al. 2015; Genz et al. 2016; Pavlidis et al. 2016; Novick et al. 2021; Wang et al. 2021; Sheludko et al. 2022; Ao et al. 2023; Menke et al. 2024; Vikhrankar et al. 2024). A noteworthy early example came from the collaboration between Merck & Co. and Codexis, which involved the introduction of 27 mutations into a class IV (*R*)-selective ω-transaminase from *Arthrobacter* sp. This successful partnership enabled large-scale biocatalytic production of the anti-diabetic drug Sitagliptin, demonstrating the potential of enzyme engineering and biocatalysis in pharmaceutical synthesis (Savile et al. 2010). Another illustration of transaminase engineering to enable interactions with bulky substrates is provided by the research conducted by Land and collaborators, in which the small binding pocket of the (*S*)-selective ω-transaminase from *Chromobacterium violaceum* was enlarged by selected mutations (Land et al. 2020).

The Sbv333-ATA is a transaminase recently discovered from a *Streptomyces* strain that shows some interesting features (Ferrandi et al. 2021). Although it comes from a mesophilic source, it is an extremely thermostable enzyme, with a melting temperature (T_m_) of 85 °C, which is only marginally lower than those of the previously reported most thermostable transaminases originating from extreme environments (87–88 °C) (Mathew et al. 2016a; Ferrandi et al. 2017). Moreover, Sbv333-ATA displays a broad substrate specificity regarding the amino acceptor spectrum and a remarkable activity in the transamination of β-ketoesters, which are rarely accepted by the other known ATAs (Ferrandi et al. 2021). Recently, Sbv333-ATA was applied, together with other amine transaminases, in the biocatalytic synthesis of vicinal amino alcohols of pharmaceutical interest, such as nor(pseudo)ephedrines (Fracchiolla et al. 2023; Patti et al. 2024).

In this paper, the properties of this interesting biocatalyst were further explored regarding its stability after exposure to organic solvents and to its specificity toward its amino donor. The high-resolution crystallographic structures of the holo and inhibitor gabaculine bound forms of native Sbv333-ATA, and holo W89A and F61C mutants were determined. These have allowed the rational design of mutant enzymes that have a broader substrate scope toward bulkier target compounds of commercial interest.

## Materials and methods

### Chemicals

Amino donors, amino acceptors, PLP, tryptone, and yeast extract were purchased from Merck Life Science (Milan, Italy). Isopropyl-β-D-thiogalactopyranoside (IPTG) was bought from VWR (Radnor, PA, USA). The remaining reagents were all of analytical grade and commercially available.

### Analytical methods

Gas chromatography (GC) analyses were carried out on a Finnigan TRACE DSQ GC/MS instrument (ThermoQuest, San Jose, CA) equipped with a HP-5MS Agilent (30 m × 0.25 mm × 0.25 μm) column, ion source 250 °C. Acetylation of amines before injection was carried out as described in (Ferrandi et al. 2017). GC analysis was carried out by keeping the column temperature at 60 °C for 1 min, then raising the temperature to 300 °C at 10 °C/min. Under these conditions, retention times of the N-acetyl derivatized amines were as follows: (*S*)-α-methylbenzylamine ((*S*)-MBA), 11.0 min; (*S*)-1-phenylpropylamine ((*S*)-PPA), 11.8 min; (*S*)-1-phenylbutylamine ((*S*)-PBA), 12.8 min; 1,2-diphenylethylamine (1,2-DPEA), 17.1 min. Retention times of the ketones were as follows: acetophenone, 6.0 min; propiophenone, 7.3 min; 2-phenylacetophenone, 14.1 min.

### Bacterial strains and plasmids

*Escherichia coli* BL21(DE3) cells harboring plasmids pETite (Lucigen, Middleton, WI, USA) coding for the Sbv333-ATA gene (GenBank accession n° WP_047470642.1) in frame with a 6x Histidine tag sequence and pGRO7 (Takara Bio Inc., Kyoto, Japan), coding for the chaperones GroES and GroEL, were available in our lab (Ferrandi et al. 2021). The gene coding for the glycine oxidase from *Geobacillus kaustophilus* HTA426 (GkGO, GenBank accession n° Q5L2C2.1) was synthesized and cloned in the pET28a vector by Twist Bioscience (South San Francisco, CA, USA). Transformation of *E. coli* BL21(DE3) cells with the above-mentioned plasmids was carried out using standard techniques. All *E. coli* strains were conserved as glycerol stocks at − 80 °C.

### Protein expression and purification

*E. coli* BL21(DE3) cells from glycerol stocks carrying plasmids coding for wild-type Sbv333-ATA, Sbv333-ATA mutants, and GkGO were placed on LB-Agar plates containing the appropriate antibiotics (kanamycin and chloramphenicol for Sbv333-ATA and kanamycin for GkGO) and incubated overnight at 37 °C. The following day, some colonies were aseptically removed by using an inoculating loop, inoculated in 100 mL of LB containing the required antibiotics, and grown for 16 h at 37 °C and 220 rpm in a thermostatic shaker (New Brunswick Innova 42, Eppendorf, Hamburg, Germany).

The pre-cultures were then used for the inoculation of 1 L of LB media, with the necessary antibiotics and 0.2 mg mL^−1^ arabinose in the case for the Sbv333-ATA expression to induce chaperone expression, and grown up at 37 °C and 220 rpm. When the OD_600_ value reached between 0.5 and 0.8, IPTG was added to a final concentration of 1 mM to stimulate the expression of the enzyme. Cell cultures were incubated at 30 °C and 220 rpm for 16 h, then cells were recovered by centrifugation at 5000 rpm and 4 °C for 30 min and resuspended in 20 mL of washing buffer (500 mM NaCl, 20 mM imidazole, 20 mM potassium phosphate (KP) buffer, pH 7.0). Cells were lysed by sonication at 0 °C (30 s-on/15 s-off × 7) and the cell lysate was centrifuged at 10,000 rpm and 4 °C for 30 min.

To perform protein purification, clear cell extracts containing the overexpressed protein were incubated with the Ni Sepharose 6 Fast Flow agarose resin (Ni–NTA) (GE Healthcare, Italy) for 60 min at 4 °C under mild shaking. The mixture was then loaded onto a glass column (10 × 110 mm), the resin was washed with 20 mL of washing buffer, then the protein was eluted using a 3-step increment (10 mL washing buffer with 100, 200, and 300 mM imidazole, respectively). Wild-type Sbv333-ATA and its mutants were dialyzed against 20 mM HEPES buffer, pH 9.0, at 4 °C for 16 h, while GkGO was dialyzed against 25 mM KP buffer, 0.2 µM FAD^+^, pH 8.5, at 4 °C for 16 h. Soluble proteins were stored at − 80 °C. Protein content was estimated according to the method of Bradford with the Bio-Rad Protein Assay and protein purity was assessed by SDS-PAGE analysis (10% T, 2.6% C). The molecular weight protein standard mixture from Bio-Rad (Karlsruhe, Germany) was used as a reference. Gels were stained for protein detection with Coomassie Brilliant Blue.

For crystallization studies, to avoid co-purification of the Sbv333-ATA with the chaperons GroES and GroEL, produced by *E. coli* BL21(DE3) carrying the plasmid pGRO7, the procedure described in the literature (Joseph and Andreotti 2008) was performed with slight modifications. Briefly, the pellet obtained after centrifugation of the cell culture was resuspended in 20 mL of a solution containing 50 mM KP buffer, pH 7.0, 500 mM NaCl, 20 mM imidazole, 5 mM ATP, and 10 mM MgCl_2_. Cells were lysed by sonication at 0 °C (30 s-on/15 s-off × 6) and the cell lysate was centrifuged at 10,000 rpm and 4 °C for 30 min. The cell extract was then incubated with Ni–NTA resin to purify the target proteins. The resin was washed with 40 mL of washing buffer, then column loading and elution of bound proteins was performed as previously described. Subsequently, to ensure a homogeneous preparation, the purified enzyme was applied to a calibrated Superdex 200 pg HiLoad 16/600 size exclusion chromatography (SEC) column (Cytiva, Marlborough, MA, US) and eluted with one column volume of 20 mM HEPES buffer, pH 9.0, 500 mM NaCl at 1.0 mL min^−1^. The purity of the protein and the subunit molecular weight were analyzed by SDS-PAGE.

### Determination of Sbv333-ATA stability in the presence of organic solvents and enzyme assays

The stability of Sbv333-ATA in organic cosolvents was evaluated by incubating the enzyme in 20 mM HEPES buffer, pH 9.0, containing 5%, 10%, and 20% (*v*/*v*) of the water-miscible cosolvents methanol, ethanol, acetonitrile (ACN), and dimethyl sulfoxide (DMSO) (final volume: 200 µL) at 25 °C and by monitoring the activity after 5 and 24 h. The stability in biphasic systems was evaluated by incubating the enzyme (20 µL of purified protein solution diluted in 80 µL of 20 mM HEPES buffer, pH 9.0) with the same volume of organic solvent (petroleum ether, toluene and ethyl acetate) at 25 °C under vigorous shaking and evaluating the activity after 5 and 24 h. Enzyme stability was monitored by using a spectrophotometric assay in quartz cuvettes (assay volume 0.8 mL) containing transaminase assay solution (2.5 mM pyruvate, 2.5 mM (*S*)-MBA in 0.1 M KP buffer, pH 9.0, and 0.25% (*v*/*v*) DMSO). Formation of acetophenone upon enzyme addition (5–20 μL of purified Sbv333-ATA) was followed at 245 nm (ε_245_ = 12 mM^−1^ cm^−1^) on a Jasco V-530 UV/VIS spectrophotometer. One unit of activity is defined as the enzyme activity that produces 1 μmol of acetophenone per minute under the assay conditions described above.

The amino donor specificity of the wild-type Sbv333-ATA and its variants was evaluated by using the glycine oxidase (GO) assay (Weiß et al. 2014). Briefly, the assay solution was prepared by dissolving 0.14 mg mL^−1^ horseradish peroxidase, 0.12 mg mL^−1^ glycine oxidase, 3 mM 4-aminoantipyrine, and 2.1 mM phenol in 50 mM HEPES buffer, pH 9.0, containing 2 mM amine donor (see Fig. 2). After the addition of the enzyme (4 µL in an assay volume of 100 µL), the increase in absorbance was followed at 500 nm (ε_500_ = 13.3 mM^−1^ cm^−1^) at room temperature. One unit of activity (U) is defined as the amount of enzyme that catalyzes the formation of one μmol of product per minute under the detailed experimental conditions described above.

### 3D-structure determination

Crystallization was carried out performing the microbatch method using an Oryx 8 automated robot (Douglas Instruments, Hungerford, UK). The protein was concentrated using a 30 kDa molecular weight cutoff Vivaspin centrifugal concentrator (Sartorius, UK) to 10 mg mL^−1^ in 20 mM HEPES, pH 9.0, 500 mM NaCl.

Initial crystallization screens were performed using Molecular Dimensions Morpheus Screens 1 and 2 and JSCG + and PACT premier. Specific crystallization conditions for each of the structures obtained are available in the Supplementary Information (Table S6). To form the crystallization droplets, 0.5 µL protein solution was added to 0.5 µL crystallization buffer, giving a final protein concentration of 5 mg mL^−1^. The droplets were covered with an 80:20 (*v*/*v*) mix of paraffin oil and silicon and kept in a vibration-free incubator at 293 K.

Diffraction data were collected to high resolution at 100 K on the Diamond Light Source synchrotron beamlines IO3 and IO4 (Table 1) using the rotation method with rotation increment of 0.1°. Data were integrated and scaled using XDS (Kabsch 2010). Structures of the holoenzyme and complexes were solved by the automated molecular replacement pipeline MORDA (Vagin and Lebedev 2015) implemented in CCP4CLOUD interface of CCP4 (Krissinel et al. 2022; Agirre et al. 2023) interface. Refinement was carried out using REFMAC5 (Murshudov et al. 2011) and dictionaries for ligands were prepared using JLIGAND (Lebedev et al. 2012).

**Table 1:** Sbv333-ATA data collection and refinement statistics

Sbv333-ATA	Native	Gabaculine	W89A	F61C
Data collection statistics				
Beamline	IO3 Diamond	IO3 Diamond	IO4 Diamond	IO4 Diamond
Wavelength (Å)	0.9763	0.9763	0.852	0.954
Space group	P21212	C2221	P1	C2221
Unit cell parameters a, b, c (Å)	78.320, 176.100, 60.850	100.463, 177.253, 112.359	63.83, 90.24, 99.45	99.747, 176.464, 111.259
α, β, γ (°)	90.0, 90.0, 90.0	90.0, 90.0,90.0	66.94, 73.26, 69.64	90.0, 90.0, 90.0
Resolution range (Å)^a^	60.85–1.49	69.59–1.31	80.02–1.24	88.23–1.31
Total reflections^a^	112,260	237,241	515,140	221,445
Unique reflections^a^	112,260 (724)	237,241 (15,399)	515,140 (506,909)	221,445 (7504)
Completeness (%)^a^	81.38 (10.66)	99.303 (92.30)	94.84 (85.04)	94.75 (64.8)
Multiplicity^a^	10.33	5 (13.3)	3.6 (3.7)	6.0 (2.9)
R_meas_ (%)^a,b^	0.043 (0.26)	0.073 (0.026)	0.071 (0.03)	0.069 (0.04)
< I >/< σ(I) >	31.8	24.2	13.7	8.9
CC_1/2_^a,c^	1 (0.9)	1 (1)	1 (1)	1 (1)
Wilson B-factor^d^ (Å^2^)	11.89	16.89	15.75	19.77
Refinement statistics				
R_work_	0.128	0.164	0.171	0.144
R_free_	0.158	0.185	0.195	0.179
No. of protein monomers in a.u	2	2	4	2
Number of atoms	8594	8717	16,920	7770
Macromolecules				
Number of protein residues	462	462	462	462
RMS bond lengths (Å)	0.0114	0.0129	0.0112	0.0137
RMS bond angles (°)	1.842	2.0140	1.849	1.967
Ramachandran favored (%)^e^	95.3	95.4	95.8	95.3
Ramachandran outliers (%)^e^	0.67	0.9	0.4	0.6
Clashscore^e^	5.12	8.7	12.7	3.3
Average B-factor protein (Å^2^)	15.4	21.3	25.3	26.9
Average B-factor ligands (Å^2^)	19.9	21.3	13.9	21.3
Average B-factor solvent (Å^2^)	25.1	31.7	56.6	45.2
RCBS PDB code	9GH9	9GNF	9QC2	9QGH

^a^Values for the highest resolution shell are given in parentheses

^b^R_meas_ = Σh [m/(m − 1)]1/2 Σi|Ih,i— < Ih >|/Σh ΣiIh,i

^c^CC1/2 is defined in Karplus and Diederichs (2012)

^d^Wilson B-factor was estimated by SFCHECK ( Vaguine et al. 1999)

^e^The Ramachandran statistics and clashscore statistics were calculated using MOLPROBITY ( Chen et al. 2010)

### Molecular docking

For the molecular docking experiments, the ligands were docked into the binding pocket designed as a 20 × 20 × 20 Å box centered at the PLP phosphate atom using AutoDock Vina as a docking engine (Trott and Oleson 2009; Eberhardt et al. 2021). The list of docking solutions was ranked by their internal scores and Gibb’s function.

### Site-directed mutagenesis

Variants of Sbv333-ATA carrying up to two-point mutations (Supplementary Information, Table S2) were generated, according to the manufacturer’s protocols, by using the Q5® Site-Directed Mutagenesis Kit (New England BioLabs, Ipswich, MA, USA) or a QuikChange II XL site-directed mutagenesis kit (Agilent, Santa Clara, CA, USA), as described in the Supplementary Information, where a list of the primers used for the mutagenesis is described (Supplementary Information, Table S1).

### Biotransformations

Transamination reactions on aromatic bulky amines with increasing side chains ((*R*)-methylbenzylamine ((*R*)-**1**), (*R*)-1-phenylpropylamine ((*R*)-**2**), (*R*)-1-phenylbutylamine ((*R*)-**3**)) were performed at 30 °C in 0.5 mL reaction mixture containing 50 mM HEPES buffer, pH 9.0 or pH 7.5, 10 mM amine, 10 mM pyruvate, 1 mM PLP, and 0.5 mg of purified enzyme.

Biotransformations on ((*S*)-methylbenzylamine ((*S*)-**1**), (*S*)-1-phenylpropylamine ((*S*)-**2**), (*S*)-1-phenylbutylamine ((*S*)-**3**), (*S*)-1,2-diphenylethylamine ((*S*)-**4**)) were carried out at 30 °C in 0.5 mL reaction mixture containing 50 mM HEPES buffer, pH 9.0, 10 mM amine, 10 mM pyruvate, 1 mM PLP, and 0.5 mg of purified enzyme. Conversions (after derivatization) were evaluated after 24 h by GC analysis as described in the “Analytical methods” section.

## Results

### Sbv333-ATA functional characterization

**Fig. 1:**
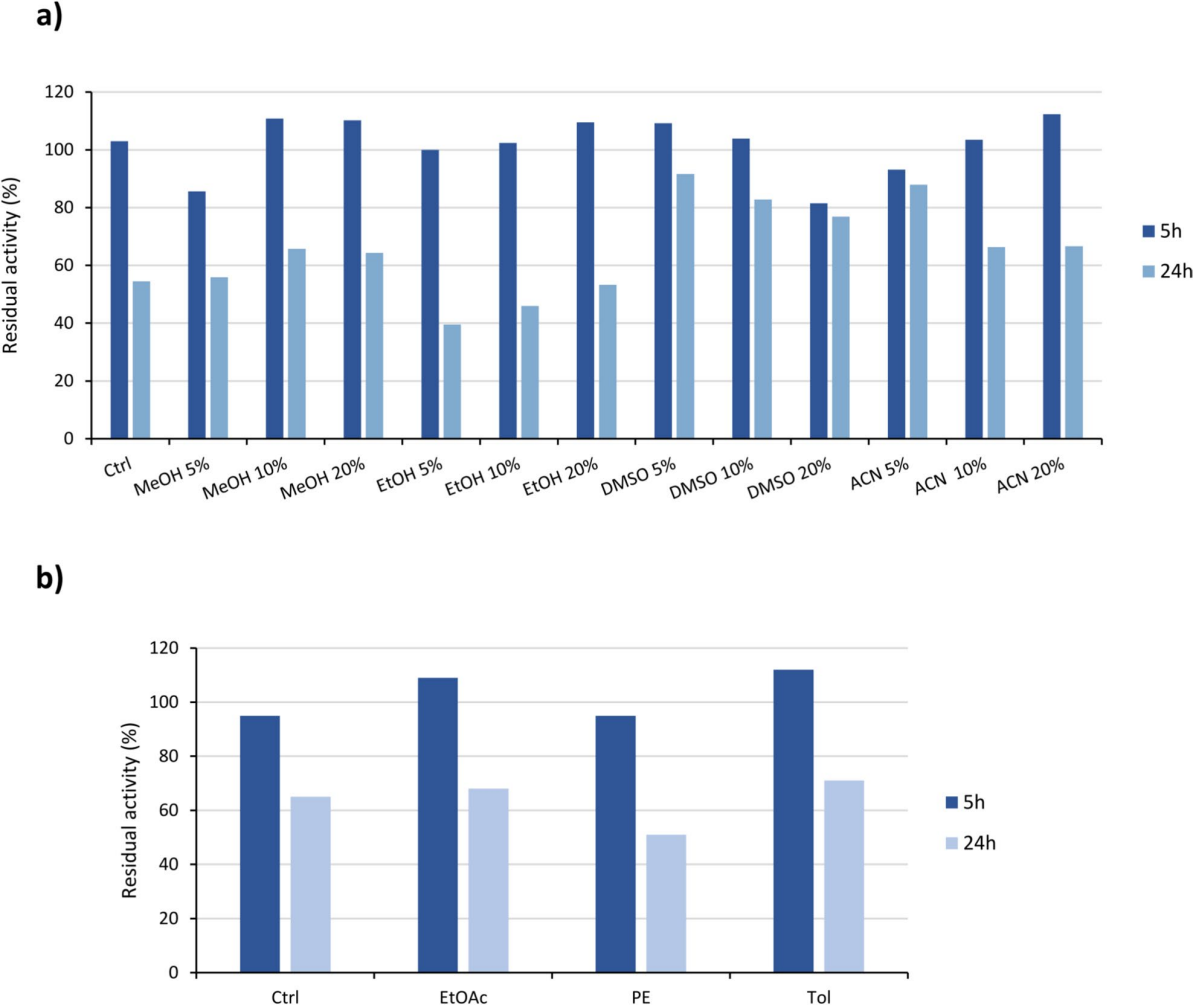
Sbv333-ATA stability to organic solvents. **a** Stability to water-miscible cosolvents, residual activity after 5 h and 24 h of incubation with 5–20% (v/v) of different solvents (methanol, MeOH; ethanol, EtOH; dimethyl sulfoxide, DMSO; acetonitrile, ACN). **b** Stability in biphasic systems (1:1), residual activity after 5 h and 24 h of incubation under shaking with ethyl acetate (EtOAc), petroleum ether (PE), and toluene (Tol) as organic phase. Residual activities were calculated considering as 100% the enzymatic activity before solvent addition (see Supplementary Information, Tables S3 and S4 for details). Reactions were performed at least in triplicate; standard deviation of residual activity was below 5%

Preliminary characterization studies demonstrated that Sbv333-ATA has a pH optimum of 9.0, it is extremely thermostable, retaining 100% of starting activity after 3 h of incubation at 70 °C and has a melting temperature of 85 °C (Ferrandi et al. 2021). The Sbv333-ATA stability was further tested in the presence of 5–20% (*v/v*) of water-miscible cosolvents: methanol, ethanol, dimethyl sulfoxide and acetonitrile. To assess the stability, the activity was evaluated using the acetophenone assay, as described in the “Materials and methods” section, at the beginning of the experiment and after 5 and 24 h. The biocatalyst proved to be stable in up to 20% (*v/v*) of organic solvent, retaining at least 40% of the starting activity after 24 h (Fig. 1a, Table S3).

The stability of Sbv333-ATA was also tested in biphasic systems (1:1, *v*/*v*), with either ethyl acetate, petroleum ether, or toluene as the organic phase. The biphasic mixtures were maintained under vigorous shaking, and the residual activity in the aqueous phase was evaluated using the acetophenone assay after 5 and 24 h. As shown in Fig. 1b (Table S4), Sbv333-ATA was tolerant to the different solvents, retaining more than 50% of its activity even after 24 h of incubation.

**Fig. 2:**
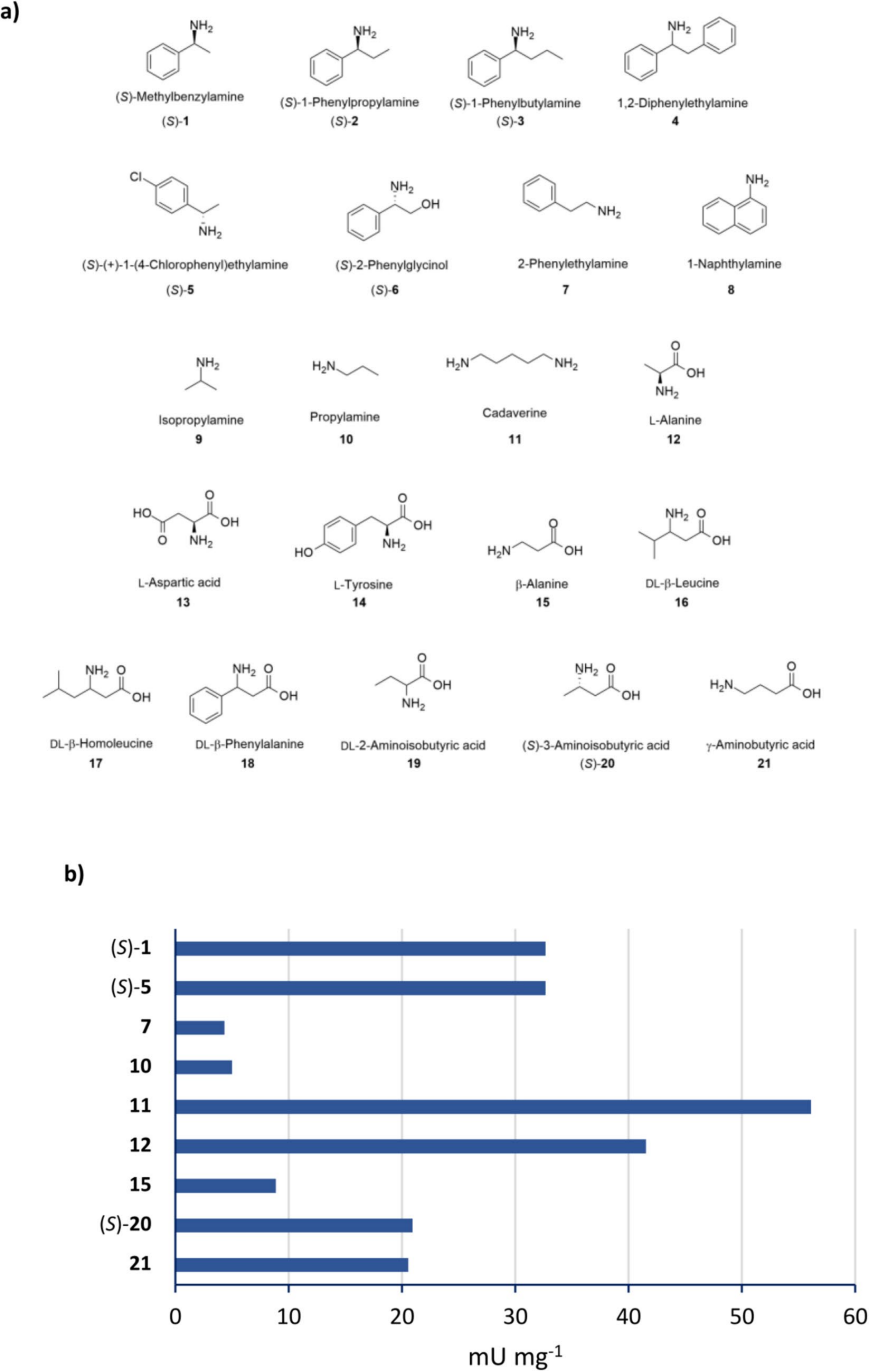
Amino donor spectrum of wild-type Sbv333-ATA. **a** Panel of screened amines; **b** Sbv333-ATA specific activity (mU mg^−1^) toward accepted amines and amino acids. Reactions were performed at least in triplicate; standard deviation of residual activity was below 5%

To evaluate the substrate scope of Sbv333-ATA, a series of aliphatic and aromatic amines, as well as different amino acids (Fig. 2a), were assayed as potential amine donors using the glycine oxidase (GO) assay (see the “Materials and methods” section for details). The enzyme was active toward a diverse set of substrates, including (*S*)-methylbenzylamine ((*S*)-**1**), 2-phenylethylamine (**7**), propylamine (**10**), cadaverine (**11**), and selected amino acids, while, as expected, more sterically hindered aromatic amines were not accepted (Fig. 2b). Negligible results were obtained with isopropyl amine, a commonly used amine donor. The substrate (*S*)-1-phenylpropylamine ((*S*)-**2**) precipitated in the assay solution, making it impossible to assess enzyme activity toward this substrate with this method.

### Sbv333-ATA 3D-structure determination and analysis

The structure of wild-type Sbv333-ATA was determined and refined to a resolution of 1.49 Å.

This enzyme crystallized as a homotetramer in the asymmetric unit. Analytical gel filtration chromatography yielded a single peak corresponding to an apparent molecular weight of 100 kDa, which would indicate a dimer. Crystals were grown without the addition of any PLP to the crystal droplet, and they grew to dimensions of 0.2 mm. A crystal of this form diffracted to 1.18 Å and belonged to the space group C 2 2 21, with the following unit cell parameters: a: 100.00, b: 176.96, c: 111.93, α = β = γ: 90.00.

**Fig. 3:**
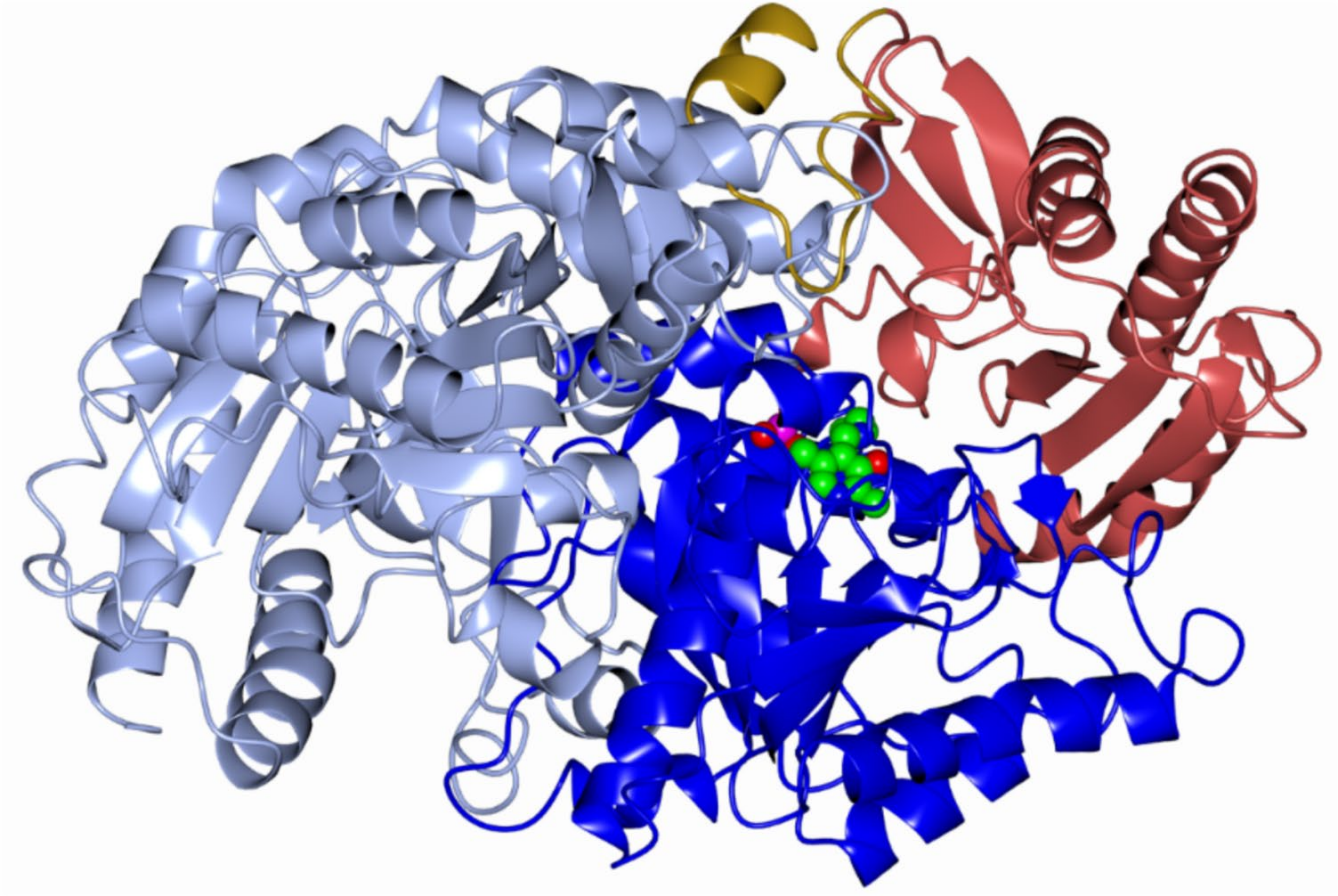
The Sbv333-ATA dimer with one monomer (right) shown with the large and small domain in dark blue and brown respectively and the PLP cofactor shown as a sphere model, the N-terminal tail and loop involved in the dimer contacts are shown in gold, and the second monomer (left) in light blue

The Sbv333-ATA monomer structure exhibits the typical transaminase type I fold (Van Oosterwijk et al. 2016), consisting of two main domains (Fig. 3). The large domain (residues 72–345) binds the PLP cofactor and contains a mostly parallel seven stranded β-sheet flanked by α-helices on both sides. The small domain comprises residues 1–71 and 346–459. The latter part contains a three-stranded antiparallel β-sheet flanked by α-helices. The N-terminal tail (residues 1–37) forms a long loop and an α-helix and is involved in the dimer contacts with the α-helix 3 and β-sheet 4 as well as a long loop consisting of residues 313–323.

**Fig. 4:**
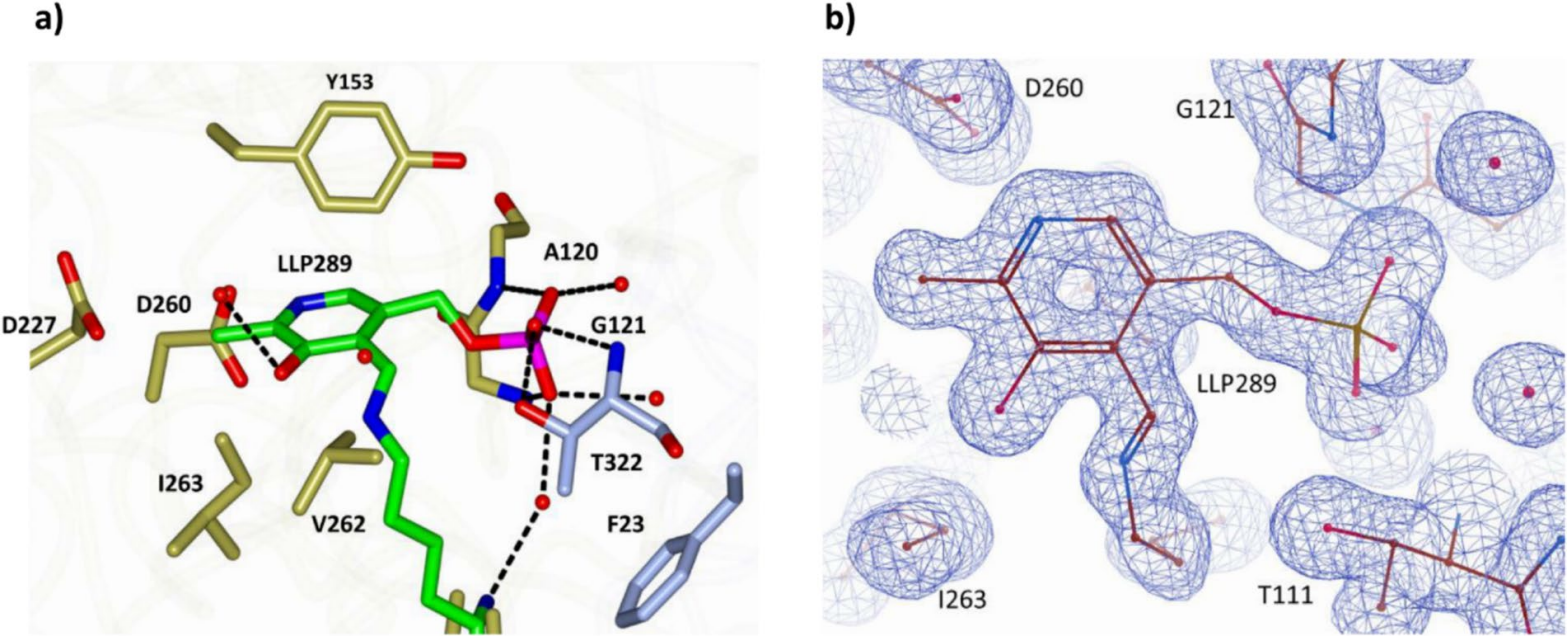
**a** The cofactor binding pocket of Sbv333-ATA, the Lys289–PLP Schiff base is highlighted as a green stick model, surrounding residues are shown in gold and light blue to discern the two opposing monomers. The dotted lines represent stabilizing hydrogen bonds. **b** Quality of the electron density at the Lys289–PLP Schiff base contoured at 1.0 σ

The PLP cofactor is covalently attached to the Lys289 side chain via a Schiff base. It binds in a pocket lined with aromatic residues (Fig. 4), with all its non-carbon atoms forming hydrogen bonds with the surrounding residues from both monomers in the dimer.

Similar to most transaminases, the Sbv333-ATA active site consists of two pockets for substrate interaction, the large (L) and the small (S) pocket. The active site cleft is formed by the two domains of one monomer and the large domain of the neighboring monomer. While residues from both subunits are involved in cofactor binding, the substrate site is primarily formed by residues from the two domains of one monomer.

### Gabaculine-inhibited complex

**Fig. 5:**
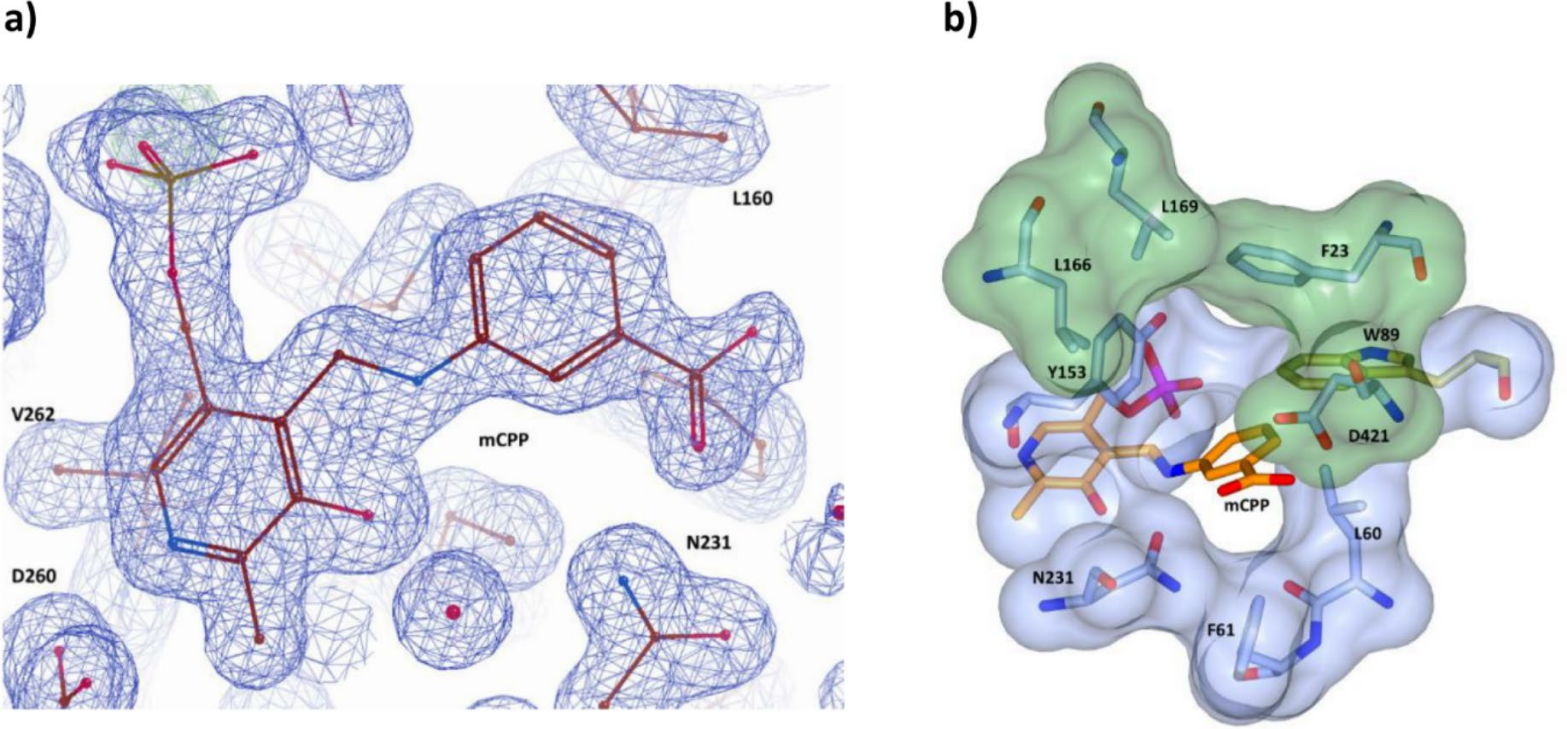
**a** Electron density map of the gabaculine complex calculated at 1.5 Å resolution. The nonhydrolysable PLP–gabaculine complex is shown as an mCPP molecule. **b** Surface view of the large pocket (green) featuring residues F23, L166, L169, and D421 depicted as stick models, and the small pocket (blue) containing residues L60, F61, Y153, N231, and W89. The mCPP molecules (orange) occupy the small pocket, leaving a visibly large empty area in the large pocket above

Gabaculine is widely recognized as a suicide inhibitor of aminotransferases. It binds to the enzyme and forms a Schiff base with the PLP cofactor. The inhibition process involves initial proton abstraction followed by a second proton removal from the adjacent carbon atom, creating an unstable intermediate. This intermediate is then converted to *m*-carboxyphenylpyridoxamine phosphate (mCPP), a highly stable compound that results in an irreversible aromatic modification of the cofactor (Rando 1977; Sayer et al. 2012). This inhibitor allows the enzyme to “freeze” in a state that resembles that of the substrate bound state. The electron density in the enzyme active site allowed modeling of the cofactor covalently bound to gabaculine as the irreversible mCPP ligand. A 2Fo-Fc electron density map for the mCPP ligand is shown in Fig. 5a.

The mCPP is positioned centrally in the active site in close proximity (< 4.5 Å) to the S pocket. The gabaculine part of the mCPP molecule is stabilized by H-bonds with N231, R419, and R423, and by hydrophobic interactions with L60 and F61, while the PLP part is bound by hydrogen bonds with G120, G121, and D260, and is held in place by hydrophobic interactions with residues Y153, H154, Q227, and V262 (Fig. S6, Supplementary Information).

On the opposite side of the active site, the L pocket outlined by residues F23, L166, L169, and D421 forms a much larger cavity which could bind the large aromatic group of substrates (Fig. 5b) (Sayer et al. 2014).

Native Sbv333-ATA has also been co-crystallized with the substrate phenylacetylcarbinol (PAC) and the product analogue norephedrine (see chemical structures in Fig. S1) (Fracchiolla et al. 2023; Patti et al. 2024). X-ray data were collected at 1.29 Å resolution for crystals of these two complexes. Electron density in both structures clearly shows two conformations of the main chain for residues 419–426, with observed displacement of Cα atom positions of up to 10 Å. One of the alternative main chain conformations exactly matches that observed in the native Sbv333-ATA structure. The other conformation differs from that observed in the gabaculine complex and has not been previously reported in any omega/amine TA structure.

The electron density of the bound ligands is partially masked by the alternative conformation of the main chain, complicating refinement of these complex structures. Despite the high concentration of co-crystallized compounds (10 mM), which should have resulted in full occupancy of the ligands in the active site, only partial occupancy was observed. This partial occupancy may result from allosteric properties of Sbv333-ATA.

### Protein engineering

The Sbv333-ATA, as demonstrated by studies shown in this paper and in our previous work (Ferrandi et al. 2021), is an enzyme with interesting features of thermostability and stability to organic solvents. Therefore, it was chosen as a starting point for the rational design of new variants with broader substrate scope, thus expanding the toolbox of stable transaminases able to interact with bulky substrates that can be used as pharmaceutical building blocks. Moreover, we also tried to reverse the enzyme’s enantioselectivity.

From the analysis of the gabaculine bound crystal structure described above, six amino acidic positions not essential for the catalytic mechanism and which contribute to the shape of the active site pockets were identified within a 5 Å radius from the bound gabaculine (F23, L60, F61, W89, Y153 and D421). Therefore, by replacing these positions with smaller amino acid residues, it is, in theory, possible to enlarge the small pocket and, in this way, allow bulky substrates to enter the active site and be converted.

Particularly, mutants L60A, L60V, W89A, W89Y, F61C, F61V, F61A, and W89A/L60V were designed with the aim of enlarging the small pocket, while mutant F23V was designed to promote bulky substrate binding by enlarging the large pocket. Mutant Y153W and the double mutants W89Y/Y153W, W89A/F23W, W89A/D421E, W89A/D421W, and W89A/F61W were designed to invert the reaction’s stereoselectivity. This could in principle be achieved by either simultaneously enlarging the small pocket and constricting the large pocket (in the case of double mutants) or by introducing a steric clash with the substrate’s aromatic ring (as with mutant Y153W) to alter enantioselectivity.

Expression trials of variants carrying up to two-point mutations were carried out, and 10 out of 14 mutants were successfully overexpressed in soluble form (see Table S2 for details).

The mutants designed to bind bulkier substrates, L60V, W89A, F61C, F61V, F23V, and W89A/L60V were characterized based on their amine donor spectrum. Screening was performed using the previously described glycine oxidase (GO) assay and the same panel of amines tested for the wild-type Sbv333-ATA (Fig. 2, Table S5). This analysis aimed to evaluate their activity on bulky substrates, such as (*S*)-1-phenylbutylamine ((*S*)-**3**) and 1,2-diphenylethylamine (**4**), while also assessing whether they retained activity on other substrates accepted by the wild-type enzyme.

**Fig. 6:**
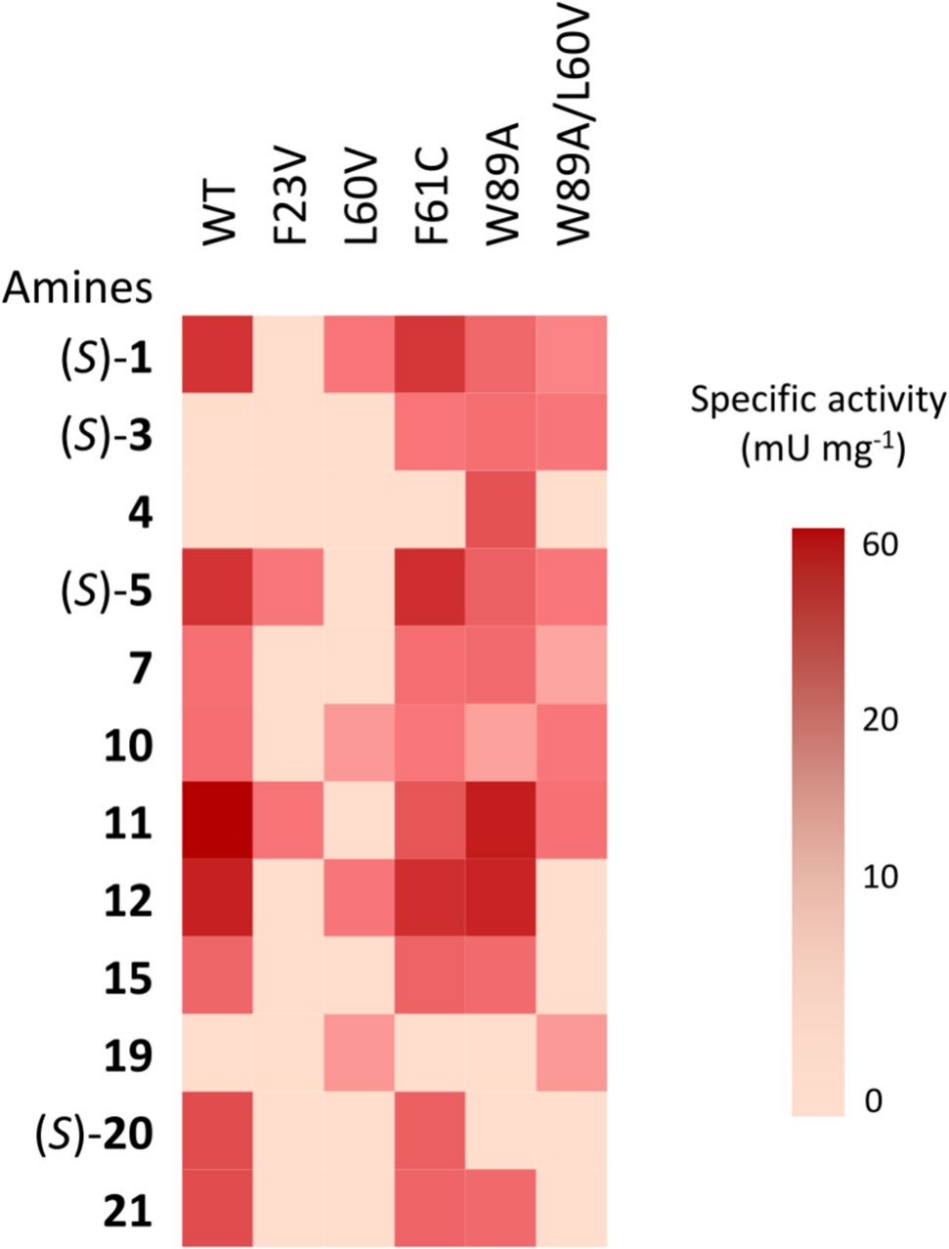
Amine donor screening of Sbv333-ATA variants. Assays were performed using the glycine oxidase (GO) assay (Weiß et al. 2014)

Under the assay conditions used, variant F61V was completely inactive, while F23V, L60V, and W89A/L60V displayed only minimal activity toward the tested substrates. However, some promising mutants with an expanded substrate scope were identified (Fig. 6, Table S5). In particular, the F61C and W89A variants retained a substrate specificity comparable to that of the wild-type enzyme while also exhibiting the ability to accept new substrates. F61C could convert substrate (*S*)-1-phenylbutylamine ((*S*)-**3**), and W89A could accept both substrates (*S*)-**3** and 1,2-diphenylethylamine (**4**).

**Fig. 7:**
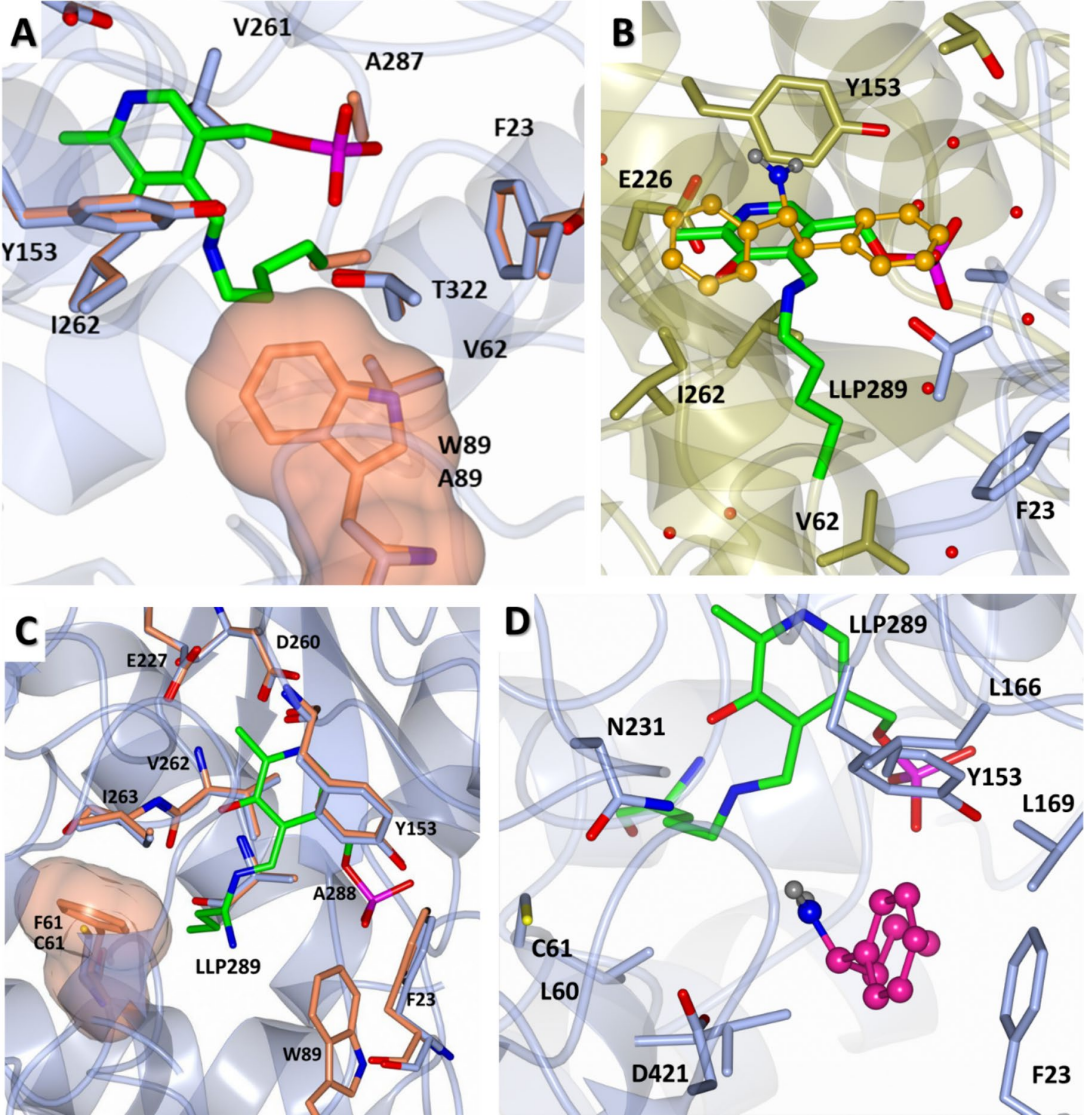
**a** Superposition of wild-type Sbv333-ATA (orange) and W89A variant (light blue) highlighting the enlargement of the S pocket resulting from the mutation of tryptophan 89 to alanine. The bulk of tryptophan 89 is shown as surface representation using the same color scheme. **b** Computational docking of 1,2-diphenylethylamine (**4**) into Sbv333-ATA W89A. The catalytically productive orientation of 1,2-diphenylethylamine is shown in orange sticks. **c** Superposition of wild-type Sbv333-ATA (orange) and F61C variant (light blue) highlighting the enlargement of the S pocket resulting from the mutation of phenylalanine 61 to cysteine, using the same color scheme as in panel **a**. **d** Computational docking of 1-phenylbutylamine ((*S*)-**3**) into Sbv333-ATA F61C. The catalytically productive orientation of 1-phenylbutylamine is shown in pink sticks

Interestingly, the structure of W89A was solved at 1.24 Å resolution and a substantial enlargement of the small pocket was observed, making the enzyme capable of accepting the bulky substrate **4**, as shown in Fig. 7a. Moreover, computational molecular docking of the aromatic substrate **4** into the active site of the W89A mutant revealed a docking pose of 1,2-diphenylethylamine in a catalytically productive orientation, in agreement with the activity data (Fig. 7b).

In a similar approach, the crystallographic structure of the F61C variant was determined at 1.31 Å resolution (Fig. 7c). The substitution of phenylalanine 61 with the smaller cysteine residue effectively reduced steric hindrance in the active site, as intended. This structural modification allowed for molecular docking of bulky phenylbutylamine ((*S*)-**3**) in multiple catalytically favorable conformations, with the lowest energy pose depicted in Fig. 7d.

In the study on stereoselectivity switching, the mutants Y153W, W89Y/Y153W, W89A/D421E, W89A/D421W, and W89A/F61W were tested against selected aromatic (*R*)-amines with increasing side chain lengths, namely, (*R*)-methylbenzylamine ((*R*)-**1**), (*R*)-1-phenylpropylamine ((*R*)-**2**), and (*R*)-1-phenylbutylamine ((*R*)-**3**) (see chemical structures in Fig. S2) using the GO assay. Unfortunately, all mutants were inactive toward these substrates.

To further assess their activity with (*S*)-amines and other amine donors, the mutants underwent a substrate screening using the GO assay with the compounds shown in Fig. 2. Mutants W89A/D421W and W89A/F61W were completely inactive with all substrates, whereas mutants W89Y/Y153W and Y153W exhibited low activity. Interestingly, the W89A/D421E variant retained full activity, displaying a substrate scope and specific activity largely comparable to that of W89A (Fig. S3).

### Biotransformation with bulky substrates

To confirm the results obtained with the GO assay with (*S*)-methylbenzylamine ((*S*)-**1**) and the bulky substrate (*S*)-1-phenylbutylamine ((*S*)-**3**), and to verify the activity toward (*S*)-1-phenylpropylamine ((*S*)-**2**) (which, as previously mentioned, precipitated in the GO assay solution), reactions with the expressed mutants were carried out on an analytical scale using pyruvate as the amine acceptor. The reactions were monitored by GC/MS analyses. The wild-type enzyme was active in the deamination of (*S*)-**2** and, as expected, demonstrated a decrease in enzyme activity when increasing the amine side chain.

**Fig. 8:**
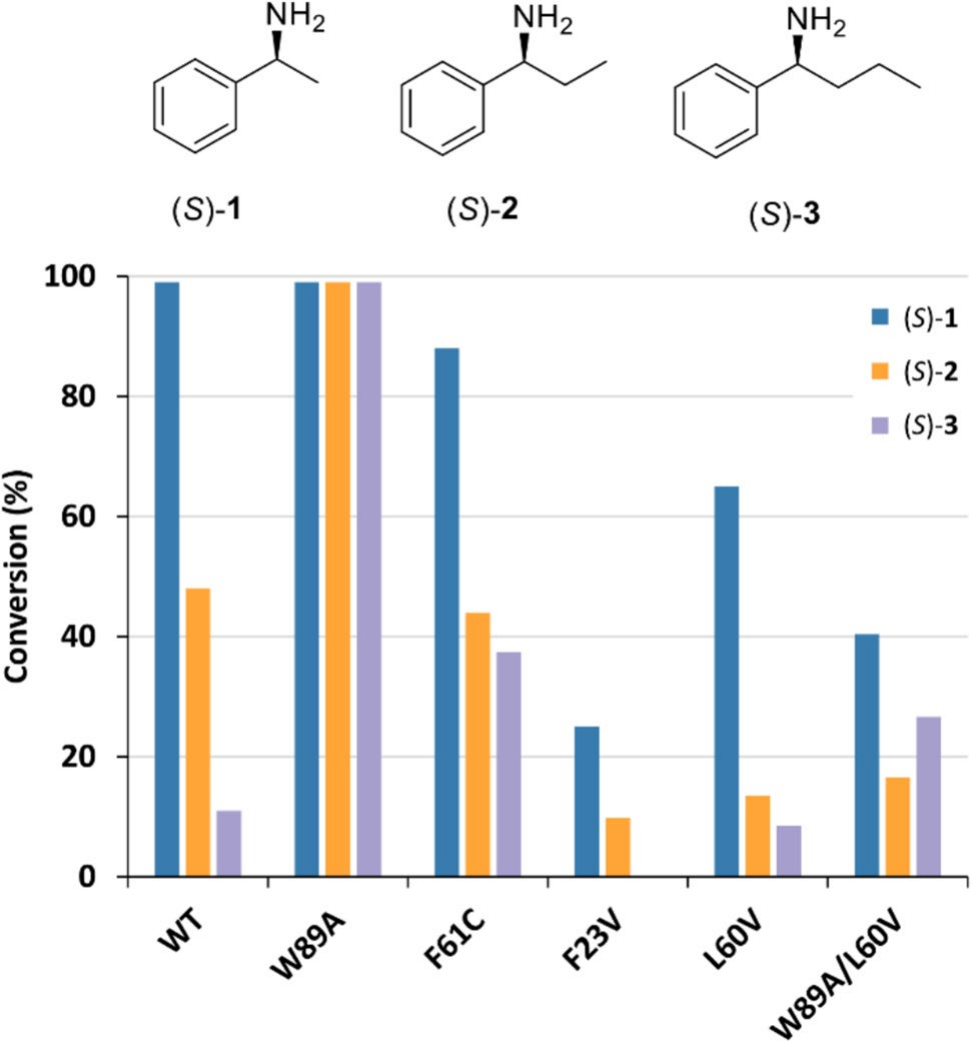
Biotransformation of selected aromatic bulky amines with increasing side chain catalyzed by wild-type Sbv333-ATA and selected mutants. Reactions were performed at least in triplicate; standard deviation of residual activity was below 5%

Unlike the results obtained with the GO assay, mutants F23V and L60V showed slight activity toward substrates (*S*)-**1** and (*S*)-**3**, respectively. In general, the other findings from the GO assay were corroborated, and among all the tested mutants, as expected, W89A stood out as the most promising, demonstrating the ability to catalyze the quantitative conversion of substrates (*S*)-**1,** (*S*)-**2** and (*S*)-**3** (Fig. 8).

Among the other mutants, F61C displayed slightly lower activity than the wild-type enzyme toward substrate (*S*)-**1**, comparable activity with substrate (*S*)-**2**, and a fourfold higher conversion with substrate (*S*)-**3**. On the other hand, the L60V mutant exhibited lower activity across all three substrates when compared to the wild type. Additionally, the double mutant W89A/L60V was less active than W89A alone.

Mutant W89A, which demonstrated activity toward the bulky-bulky substrate 1,2-diphenylethylamine (**4**) in the colorimetric screening, was also tested in small-scale biotransformation with optically pure (*S*)-**4**. Remarkably, it exhibited high substrate conversion (> 90%), confirming its activity on this sterically hindered molecule.

Finally, mutant W89A was tested in small-scale biotransformation of (*R*)-amines ((*R*)-methylbenzylamine ((*R*)-**1**), (*R*)-1-phenylpropylamine ((*R*)-**2**), (*R*)-1-phenylbutylamine ((*R*)-**3**), and (*R*)-1,2-diphenylethylamine ((*R*)-**4**), see chemical structures in Fig. S4) to verify its stereospecificity. Considering that for the wild-type enzyme, increased pH values were associated with enhanced conversion rates, but at the expense of enantioselectivity (Patti et al. 2024), reactions were performed at pH 9.0 or at pH 7.5 for comparison. As shown in Fig. S4, the W89A variant showed low conversions at pH 9.0 (< 20%), while at pH 7.5 the enzyme exhibited higher stereoselectivity, with only some residual activity on (*R*)-**1**. Thus, the trend observed with the W89A mutant aligns with our previous findings on the wild-type enzyme, i.e., confirming its (*S*) stereospecificity and highlighting the effect of pH in the outcome of (*R*)-amines deamination reactions.

## Discussion

The engineering of transaminases has emerged as a powerful strategy in the development of sustainable and selective biocatalytic processes for the synthesis of pharmaceutical intermediates and active ingredients (Vikhrankar et al. 2024). These enzymes have proven to be highly versatile tools for asymmetric synthesis due to their ability to operate under mild conditions, their inherent stereoselectivity, and the possibility to tailor their substrate scope and stability through protein engineering (Cui et al. 2024). In recent years, advances in structure-guided design and high-throughput screening have significantly expanded the applicability of these biocatalysts, enabling the efficient transformation of increasingly complex and bulky amine substrates (Savile et al. 2010; Ma et al. 2022; Prier et al. 2023). Within this framework, our study focuses on Sbv333-ATA, a wild-type transaminase with promising characteristics for further development as a biocatalyst relevant to pharmaceutical synthesis.

Sbv333-ATA is a recently identified transaminase from *Streptomyces* sp., demonstrating noteworthy features, including thermostability and broad substrate specificity in terms of its amine acceptor spectrum (Ferrandi et al. 2021).

These intriguing properties prompted further exploration and functional characterization of this biocatalyst. Our investigation assessed stability in organic solvents and expanded the known amine acceptor spectrum. Additionally, we determined the high-resolution crystallographic structure of the enzyme, enabling detailed analysis of the active site and rational mutagenesis to engineer accommodation of bulkier substrates.

Studying the stability of this enzyme in organic solvents is particularly intriguing, as synthetic reactions frequently rely on such solvents. This presents a key challenge in biocatalysis, as enzymes are typically optimized for aqueous environments and often exhibit limited stability when exposed to organic solvents.

However, the solubility of ketones in water is often quite low, making an otherwise straightforward substrate addition process more difficult. In these cases, the use of a cosolvent is necessary, the most widely used of which is certainly dimethyl sulfoxide, at least at the screening level, and this explains the need for robust enzymes capable of remaining stable even in the presence of such organic solvents (Madsen and Woodley 2023).

In this regard, Sbv333-ATA has exhibited favorable outcomes, demonstrating it to be stable even in the presence of 20% (*v*/*v*) water-miscible cosolvents (MeOH, EtOH, DMSO, and ACN), or in biphasic systems (EtOAc, PE, Tol). It retained nearly all its initial activity after 5 h of incubation and at least 40% of the initial activity after 24 h. This characteristic is crucial from an industrial applicability standpoint, where stability in organic solvents is necessary for many synthetic processes to improve the substrate’s solubility. To the best of our knowledge, there are numerous examples of engineered transaminases showing an enhanced stability (Meng et al. 2022), but few wild-type transaminases are capable of operating in organic solvents. In particular, solvent tolerance of Sbv333-ATA resembles well, if not exceeds, that of recently discovered (hyper)thermostable transaminases from different sources (Mathew et al. 2016b, 2016a; Ferrandi et al. 2017; Márquez et al. 2019). Moreover, unlike other ATAs showing a significant decrease in enzyme activity immediately after cosolvents addition (Cerioli et al. 2015), this effect was generally not observed with Sbv333-ATA. Instead, small amounts of some cosolvents, e.g., 5% (*v*/*v*) DMSO or ACN, would seem to have almost a stabilizing effect (around 90% residual activity after 24 h vs. 54% in the control sample).

The amine donor spectrum was then evaluated. Among the tested substrates, the enzyme demonstrated activity toward various compounds, including aromatic amines such as (*S*)-methylbenzylamine and 2-phenylethylamine, aliphatic mono- or di-amines like propylamine and cadaverine, and specific amino acids including β-alanine and γ-aminobutyric acid (not accepted by the largely employed ATAs from *C. violaceum* and *V. fluvialis*). As assumed, aromatic amines with greater steric hindrance were not accommodated, consistent with numerous wild-type transaminases (Ferrandi and Monti 2018). However, given the substantial industrial interest in bulkier substrates and considering the intriguing stability results demonstrated by this enzyme, we attempted to broaden the substrate scope through protein engineering to generate mutants with improved features.

The determination of the enzyme’s three-dimensional structure has served as the starting point for a rational design of variants with an expanded catalytic site, allowing the enzyme to accommodate non-natural and bulkier substrates. The catalytic site of transaminases is characterized by two pockets accommodating the substrate: a small pocket and a large one. Our efforts have focused on enlarging the small pocket.

From the study of the homology sequence alignment reinforced by the analysis of native and gabaculine bound structures, the catalytic site was identified precisely. Thus, we used structure-guided rational design to identify numerous potential residues for mutation among the non-essential amino acids for catalytic activity that contribute to steric hindrance. Specifically, 10 variants were successfully expressed with yields comparable to or slightly lower than the wild-type enzyme. All these variants were characterized by evaluating their substrate scope differences.

According to the colorimetric GO assay, variants W89A, F61C, and W89A/L60V demonstrated the ability to accept bulkier substrates such as (*S*)-phenylbutylamine ((*S*)-**3**). Additionally, W89A was able to accommodate the bulky-bulky molecule 1,2-diphenylethylamine (**4**). Overall, the mutation of residue W89 significantly reduced the steric hindrance in the active site S pocket, expanding substrate specificity.

Given the extensive literature available on transaminase enzyme engineering, we have compared the amino acids identified in our study with those already mutated in other transaminases. Structural alignment (Fig. S5) was performed, revealing that certain mutable positions on our protein corresponded to hot spots previously mutated in other enzymes*.* For example, the mutations at position F19, W57, and F85 in the TA from *V. fluvialis* (Midelfort et al. 2013; Novick et al. 2021), L57 in the ω-TA from *Ochrobactrum anthropi* (Han et al. 2015), and V153 in the ω-TA from *Paracoccus denitrificans* (Park et al. 2014) have allowed for an expansion of the active site and align with the residues F23, L60, W89, and Y153 mutated by us in this study. Similarly, mutations Y87 and Y152 were all identified as some of the best substitutions for improvement of the conversion of bulky substrates in *Silicibacter* sp. TA (Ao et al. 2023; Menke et al. 2024); these mutations corresponding to W89 and Y153 in this study. However, it is remarkable that, in our work, the aim of the Y153 substitution was to induce a selectivity switch by increasing steric hindrance with the bulky tryptophan residue.

The mutants exhibiting the highest potential were tested in the biotransformation of aromatic amines. Beyond confirming most of the findings from the prior screening, it was observed that the W89A mutant effectively catalyzed the desired biotransformation, achieving quantitative conversions of aromatic (*S*)-amines with increasing side chains (> 99%). Moreover, this mutant demonstrated the ability to maintain its stereoselectivity, in particular by showing either none or negligible conversion of the corresponding (*R*)-amines at pH 7.5.

Notably, W89A/V60L was demonstrated to be less active than W89A in the deamination of substrates (*S*)-**1**–**3**, which is likely because of its even larger binding pocket that creates excessive space for bulky-bulky substrates, preventing them from being properly positioned in the active site for catalysis. In addition, the F61C mutant was active in the deamination of (*S*)-**3**, achieving conversion levels that were significantly higher than those of the wild-type enzyme.

In conclusion, Sbv333-ATA represents a highly promising transaminase with exceptional stability and a broad amine acceptor spectrum. Its ability to retain activity in the presence of organic cosolvents and biphasic systems makes it a valuable candidate for industrial biocatalysis. Furthermore, the successful engineering of variants, particularly W89A, has significantly expanded its substrate scope to accommodate bulkier molecules while maintaining high activity and stereoselectivity. These findings not only enhance our understanding of transaminase function but also provide a strong foundation for future enzyme engineering efforts aimed at optimizing biocatalysts for industrial applications.

## Supplementary Information

Below is the link to the electronic supplementary material.

ESM 1 (DOCX 916 KB)

## Data Availability

Crystallographic Protein Structure data has been deposited in the Protein Data Base. Data is provided within the manuscript or Supplementary information files. Details of the biotransformation data will be deposited in the University of Exeter ORE repository after acceptance of the manuscript.

## References

[CR1] Agirre J, Atanasova M, Bagdonas H, Ballard CB, Baslé A, Beilsten-Edmands J, Borges RJ, Brown DG, Burgos-Mármol JJ, Berrisford JM, Bond PS, Caballero I, Catapano L, Chojnowski G, Cook AG, Cowtan KD, Croll TI, Debreczeni J, Devenish NE, Dodson EJ, Drevon TR, Emsley P, Evans G, Evans PR, Fando M, Foadi J, Fuentes-Montero L, Garman EF, Gerstel M, Gildea RJ, Hatti K, Hekkelman ML, Heuser P, Hoh SW, Hough MA, Jenkins HT, Jiménez E, Joosten RP, Keegan RM, Keep N, Krissinel EB, Kolenko P, Kovalevskiy O, Lamzin VS, Lawson DM, Lebedev AA, Leslie AGW, Lohkamp B, Long F, Malý M, McCoy AJ, McNicholas SJ, Medina A, Millán C, Murray JW, Murshudov GN, Nicholls RA, Noble MEM, Oeffner R, Pannu NS, Parkhurst JM, Pearce N, Pereira J, Perrakis A, Powell HR, Read RJ, Rigden DJ, Rochira W, Sammito M, Rodríguez FS, Sheldrick GM, Shelley KL, Simkovic F, Simpkin AJ, Skubak P, Sobolev E, Steiner RA, Stevenson K, Tews I, Thomas JMH, Thorn A, Valls JT, Uski V, Usón I, Vagin A, Velankar S, Vollmar M, Walden H, Waterman D, Wilson KS, Winn MD, Winter G, Wojdyr M, Yamashita K (2023) The CCP4 suite: integrative software for macromolecular crystallography. Acta Crystallogr D Struct Biol 79:449–461. 10.1107/S2059798323003595 37259835 10.1107/S2059798323003595 PMC10233625

[CR2] Alcántara AR, Domínguez de María P, Littlechild JA, Schürmann M, Sheldon RA, Wohlgemuth R (2022) Biocatalysis as key to sustainable industrial chemistry. Chemsuschem 15:e202200709. 10.1002/cssc.202200709 35445559 10.1002/cssc.202200709

[CR3] Ao Y, Pei S, Xiang C, Menke MJ, Shen L, Sun C, Dörr M, Born S, Höhne M, Bornscheuer UT (2023) Structure‐ and data‐driven protein engineering of transaminases for improving activity and stereoselectivity. Angew Chem Int Ed 62:e202301660. 10.1002/anie.202301660 10.1002/anie.202301660 37022103

[CR4] Bornscheuer UT, Huisman GW, Kazlauskas RJ, Lutz S, Moore JC, Robins K (2012) Engineering the third wave of biocatalysis. Nature 485:185–194. 10.1038/nature11117 22575958 10.1038/nature11117

[CR5] Buller R, Lutz S, Kazlauskas RJ, Snajdrova R, Moore JC, Bornscheuer UT (2023) From nature to industry: harnessing enzymes for biocatalysis. Science (1979) 382:eadh8615. 10.1126/science.adh8615 10.1126/science.adh8615 37995253

[CR6] Cerioli L, Planchestainer M, Cassidy J, Tessaro D, Paradisi F (2015) Characterization of a novel amine transaminase from *Halomonas elongata*. J Mol Catal B Enzym 120:141–150. 10.1016/j.molcatb.2015.07.009

[CR7] Chen VB, Arendall WB, Headd JJ, Keedy DA, Immormino RM, Kapral GJ, Murray LW, Richardson JS, Richardson DC (2010) MolProbity : all-atom structure validation for macromolecular crystallography. Acta Crystallogr D Biol Crystallogr 66:12–21. 10.1107/S0907444909042073 20057044 10.1107/S0907444909042073 PMC2803126

[CR8] Constable DJCC, Dunn PJ, Hayler JD, Humphrey GR, Leazer JL Jr, Linderman RJ, Lorenz K, Manley J, Pearlman BA, Wells A, Zaks A, Zhang TY (2007) Key green chemistry research areas—a perspective from pharmaceutical manufacturers. Green Chem 9:411–420. 10.1039/B703488C

[CR9] Cui Y, Gao Y, Yang L (2024) Transaminase catalyzed asymmetric synthesis of active pharmaceutical ingredients. Green Synth Catal.10.1016/j.gresc.2024.03.003

[CR10] Eberhardt J, Santos-Martins D, Tillack AF, Forli S (2021) AutoDock Vina 1.2.0: new docking methods, expanded force field, and python bindings. J Chem Inf Model 61:3891–3898. 10.1021/acs.jcim.1c00203 34278794 10.1021/acs.jcim.1c00203 PMC10683950

[CR11] Ferrandi EE, Monti D (2018) Amine transaminases in chiral amines synthesis: recent advances and challenges. World J Microbiol Biotechnol 34:13. 10.1007/s11274-017-2395-2 10.1007/s11274-017-2395-2 29255954

[CR12] Ferrandi EE, Previdi A, Bassanini I, Riva S, Peng X, Monti D (2017) Novel thermostable amine transferases from hot spring metagenomes. Appl Microbiol Biotechnol 101:4963–4979. 10.1007/s00253-017-8228-2 28357542 10.1007/s00253-017-8228-2

[CR13] Ferrandi EE, Spasic J, Djokic L, Vainshtein Y, Senthamaraikannan R, Vojnovic S, Grumaz C, Monti D, Nikodinovic-Runic J (2021) Novel Transaminase and Laccase from *Streptomyces* spp. Using combined identification approaches. Catalysts 11:919. 10.3390/catal11080919

[CR14] Fracchiolla N, Patti S, Sangalli F, Monti D, Presini F, Giovannini PP, Parmeggiani F, Brenna E, Tessaro D, Ferrandi EE (2023) Insight into the stereoselective synthesis of (1*S*)‐Nor(pseudo)ephedrine analogues by a two‐steps biocatalytic process. ChemCatChem e202301199. 10.1002/cctc.202301199

[CR15] Fuchs M, Farnberger JE, Kroutil W (2015) The industrial age of biocatalytic transamination. Eur J Org Chem 2015:6965–6982. 10.1002/ejoc.201500852 10.1002/ejoc.201500852 PMC4690199 26726292

[CR16] Genz M, Melse O, Schmidt S, Vickers C, Dörr M, van den Bergh T, Joosten H-J, Bornscheuer UT (2016) Engineering the amine transaminase from *Vibrio fluvialis* towards branched-chain substrates. ChemCatChem 8:3199–3202. 10.1002/cctc.201601007

[CR17] Genz M, Vickers C, van den Bergh T, Joosten H-J, Dörr M, Höhne M, Bornscheuer U (2015) Alteration of the donor/acceptor spectrum of the (*S*)-Amine Transaminase from *Vibrio fluvialis*. Int J Mol Sci 16:26953–26963. 10.3390/ijms161126007 26569229 10.3390/ijms161126007 PMC4661865

[CR18] Ghislieri D, Turner NJ (2014) Biocatalytic approaches to the synthesis of enantiomerically pure chiral amines. Top Catal 57:284–300. 10.1007/s11244-013-0184-1

[CR19] Guo F, Berglund P (2017) Transaminase biocatalysis: optimization and application. Green Chem 19:333–360. 10.1039/C6GC02328B

[CR20] Han S, Park E, Dong J, Shin J (2015) Expanding substrate specificity of ω-Transaminase by rational remodeling of a large substrate-binding pocket. Adv Synth Catal 357:2712–2720. 10.1002/adsc.201500239

[CR21] Hughes DL (2018) Biocatalysis in drug development—Highlights of the recent patent literature. Org Process Res Dev 22:1063–1080. 10.1021/acs.oprd.8b00232

[CR22] Isupov MN, Boyko KM, Sutter J-M, James P, Sayer C, Schmidt M, Schönheit P, Nikolaeva AYu, Stekhanova TN, Mardanov AV, Ravin NV, Bezsudnova EYu, Popov VO, Littlechild JA (2019) Thermostable branched-chain amino acid transaminases from the Archaea *Geoglobus acetivorans* and *Archaeoglobus fulgidus*: biochemical and structural characterization. Front Bioeng Biotechnol 7:1–16. 10.3389/fbioe.2019.00007 30733943 10.3389/fbioe.2019.00007 PMC6353796

[CR23] Joseph RE, Andreotti AH (2008) Bacterial expression and purification of Interleukin-2 Tyrosine kinase: single step separation of the chaperonin impurity. Protein Expr Purif 60:194–197. 10.1016/j.pep.2008.04.001 18495488 10.1016/j.pep.2008.04.001 PMC2581883

[CR24] Kabsch W (2010) XDS. Acta Crystallogr D Biol Crystallogr 66:125–132. 10.1107/S0907444909047337 20124692 10.1107/S0907444909047337 PMC2815665

[CR25] Karplus PA, Diederichs K (2012) Linking crystallographic model and data quality. M&M, supporting info. Science 336:1030–1033. 10.1126/science.1218231 22628654 10.1126/science.1218231 PMC3457925

[CR26] Kelly SA, Pohle S, Wharry S, Mix S, Allen CCR, Moody TS, Gilmore BF (2018) Application of ω-Transaminases in the pharmaceutical industry. Chem Rev 118:349–367. 10.1021/acs.chemrev.7b00437 29251912 10.1021/acs.chemrev.7b00437

[CR27] Krissinel E, Lebedev AA, Uski V, Ballard CB, Keegan RM, Kovalevskiy O, Nicholls RA, Pannu NS, Skubák P, Berrisford J, Fando M, Lohkamp B, Wojdyr M, Simpkin AJ, Thomas JMH, Oliver C, Vonrhein C, Chojnowski G, Basle A, Purkiss A, Isupov MN, Mcnicholas S, Lowe E, Trivinõ J, Cowtan K, Agirre J, Rigden DJ, Uson I, Lamzin V, Tews I, Bricogne G, Leslie AGW, Brown DG, Antonyuk S (2022) CCP4 Cloud for structure determination and project management in macromolecular crystallography. Acta Crystallogr D Struct Biol 78:1079–1089. 10.1107/S2059798322007987 36048148 10.1107/S2059798322007987 PMC9435598

[CR28] Land H, Ruggieri F, Szekrenyi A, Fessner W, Berglund P (2020) Engineering the active site of an (*S*)-Selective amine transaminase for acceptance of doubly bulky primary amines. Adv Synth Catal 362:812–821. 10.1002/adsc.201901252

[CR29] Lebedev AA, Young P, Isupov MN, Moroz OV, Vagin AA, Murshudov GN (2012) JLigand: A graphical tool for the CCP4 template-restraint library. Acta Crystallogr D Biol Crystallogr 68:431–440. 10.1107/S090744491200251X 22505263 10.1107/S090744491200251X PMC3322602

[CR30] Łyskowski A, Gruber C, Steinkellner G, Schürmann M, Schwab H, Gruber K, Steiner K (2014) Crystal Structure of an (*R*)-Selective ω-Transaminase from *Aspergillus terreus*. PLoS ONE 9:e87350. 10.1371/journal.pone.0087350 24498081 10.1371/journal.pone.0087350 PMC3907554

[CR31] Ma Y, Jiao X, Wang Z, Mu H, Sun K, Li X, Zhao T, Liu X, Zhang N (2022) Engineering a transaminase for the efficient synthesis of a key intermediate for rimegepant. Org Process Res Dev 26:1971–1977. 10.1021/acs.oprd.1c00376

[CR32] Madsen JØ, Woodley JM (2023) Considerations for the scale-up of in vitro transaminase-catalyzed asymmetric synthesis of chiral amines. ChemCatChem 15:e202300560. 10.1002/cctc.202300560

[CR33] Márquez SL, Atalah J, Blamey JM (2019) Characterization of a novel thermostable (*S*)-amine-transaminase from an Antarctic moderately-thermophilic bacterium *Albidovulum *sp. SLM16. Enzyme Microb Technol 131:109423. 10.1016/j.enzmictec.2019.109423 10.1016/j.enzmictec.2019.109423 31615676

[CR34] Mathew S, Deepankumar K, Shin G, Hong EY, Kim B-G, Chung T, Yun H (2016a) Identification of novel thermostable ω-transaminase and its application for enzymatic synthesis of chiral amines at high temperature. RSC Adv 6:69257–69260. 10.1039/C6RA15110H

[CR35] Mathew S, Nadarajan SP, Chung T, Park HH, Yun H (2016b) Biochemical characterization of thermostable ω-transaminase from *Sphaerobacter thermophilus* and its application for producing aromatic β- and γ-amino acids. Enzyme Microb Technol 87–88:52–60. 10.1016/j.enzmictec.2016.02.013 27178795 10.1016/j.enzmictec.2016.02.013

[CR36] Mehta PK, Hale TI, Christen P (1993) Aminotransferases: demonstration of homology and division into evolutionary subgroups. Eur J Biochem 214:549–561. 10.1111/j.1432-1033.1993.tb17953.x 8513804 10.1111/j.1432-1033.1993.tb17953.x

[CR37] Meng Q, Ramírez-Palacios C, Wijma HJ, Janssen DB (2022) Protein engineering of amine transaminases. Front Catal 2:1–28. 10.3389/fctls.2022.1049179

[CR38] Menke MJ, Ao YF, Bornscheuer UT (2024) Practical machine learning-assisted design protocol for protein engineering: transaminase engineering for the conversion of bulky substrates. ACS Catal 14:6462–6469. 10.1021/acscatal.4c00987

[CR39] Midelfort KS, Kumar R, Han S, Karmilowicz MJ, McConnell K, Gehlhaar DK, Mistry A, Chang JS, Anderson M, Villalobos A, Minshull J, Govindarajan S, Wong JW (2013) Redesigning and characterizing the substrate specificity and activity of *Vibrio fluvialis* aminotransferase for the synthesis of imagabalin. Protein Eng des Sel 26:25–33. 10.1093/protein/gzs065 23012440 10.1093/protein/gzs065

[CR40] Murshudov GN, Skubák P, Lebedev AA, Pannu NS, Steiner RA, Nicholls RA, Winn MD, Long F, Vagin AA (2011) REFMAC5 for the refinement of macromolecular crystal structures. Acta Crystallogr D Biol Crystallogr 67:355–367. 10.1107/S0907444911001314 21460454 10.1107/S0907444911001314 PMC3069751

[CR41] Novick SJ, Dellas N, Garcia R, Ching C, Bautista A, Homan D, Alvizo O, Entwistle D, Kleinbeck F, Schlama T, Ruch T (2021) Engineering an amine transaminase for the efficient production of a chiral sacubitril precursor. ACS Catal 11:3762–3770. 10.1021/acscatal.0c05450

[CR42] Park E, Park S, Han S, Dong J, Shin J (2014) Structural determinants for the non-canonical substrate specificity of the ω-Transaminase from *Paracoccus denitrificans*. Adv Synth Catal 356:212–220. 10.1002/adsc.201300786

[CR43] Patti S, Magrini Alunno I, Semproli R, Tessaro D, Monti D, Riva S, Ubiali D, Ferrandi EE (2024) Two‐step biocatalytic synthesis of (1*S*)‐Nor(pseudo)ephedrine catalyzed by immobilized enzymes. ChemCatChem e202400861. 10.1002/cctc.202400861

[CR44] Pavlidis IV, Weiß MS, Genz M, Spurr P, Hanlon SP, Wirz B, Iding H, Bornscheuer UT (2016) Identification of (*S*)-selective transaminases for the asymmetric synthesis of bulky chiral amines. Nat Chem 8:1076–1082. 10.1038/nchem.2578 27768108 10.1038/nchem.2578

[CR45] Prier CK, Camacho Soto K, Forstater JH, Kuhl N, Kuethe JT, Cheung-Lee WL, Di Maso MJ, Eberle CM, Grosser ST, Ho H-I, Hoyt E, Maguire A, Maloney KM, Makarewicz A, McMullen JP, Moore JC, Murphy GS, Narsimhan K, Pan W, Rivera NR, Saha-Shah A, Thaisrivongs DA, Verma D, Wyatt A, Zewge D (2023) Amination of a green solvent via immobilized biocatalysis for the synthesis of nemtabrutinib. ACS Catal 13:7707–7714. 10.1021/acscatal.3c00941

[CR46] Rando RR (1977) Mechanism of the irreversible inhibition of γ-aminobutyric acid-α-ketoglutaric acid transaminase by the neurotoxin gabaculine. Biochemistry 16:4604–4610. 10.1021/bi00640a012 410442 10.1021/bi00640a012

[CR47] Savile CK, Janey JM, Mundorff EC, Moore JC, Tam S, Jarvis WR, Colbeck JC, Krebber A, Fleitz FJ, Brands J, Devine PN, Huisman GW, Hughes GJ (2010) Biocatalytic asymmetric synthesis of chiral amines from ketones applied to sitagliptin manufacture. Science 329:305–309. 10.1126/science.1188934 20558668 10.1126/science.1188934

[CR48] Sayer C, Bommer M, Isupov M, Ward J, Littlechild J (2012) Crystal structure and substrate specificity of the thermophilic serine:pyruvate aminotransferase from *Sulfolobus solfataricus*. Acta Crystallogr D Biol Crystallogr 68:763–772. 10.1107/S0907444912011274 22751661 10.1107/S0907444912011274

[CR49] Sayer C, Isupov MN, Westlake A, Littlechild JA (2013) Structural studies of *Pseudomonas* and *Chromobacterium* ω-aminotransferases provide insights into their differing substrate specificity. Acta Crystallogr D Biol Crystallogr 69:564–576. 10.1107/S0907444912051670 23519665 10.1107/S0907444912051670 PMC3606037

[CR50] Sayer C, Martinez-Torres RJ, Richter N, Isupov MN, Hailes HC, Littlechild JA, Ward JM (2014) The substrate specificity, enantioselectivity and structure of the (*R*)-selective amine: pyruvate transaminase from *Nectria haematococca*. FEBS J 281:2240–2253. 10.1111/febs.12778 24618038 10.1111/febs.12778 PMC4255305

[CR51] Sheludko YV, Slagman S, Gittings S, Charnock SJ, Land H, Berglund P, Fessner W (2022) Enantioselective synthesis of pharmaceutically relevant bulky arylbutylamines using engineered transaminases. Adv Synth Catal 364:2972–2981. 10.1002/adsc.202200403

[CR52] Trott O, Oleson AJ (2009) Software news and update AutoDock Vina: improving the speed and accuracy of docking with a new scoring function, efficient optimization, and multithreading. J Comput Chem 455–461. 10.1002/jcc 10.1002/jcc.21334 PMC3041641 19499576

[CR53] Vagin A, Lebedev A (2015) MoRDa, an automatic molecular replacement pipeline. Acta Crystallogr A 71:s19. 10.1107/S2053273315099672

[CR54] Vaguine AA, Richelle J, Wodak SJ (1999) SFCHECK: a unified set of procedures for evaluating the quality of macromolecular structure-factor data and their agreement with the atomic model. Acta Crystallogr D Biol Crystallogr 55:191–205. 10.1107/S0907444998006684 10089410 10.1107/S0907444998006684

[CR55] Van Oosterwijk N, Willies S, Hekelaar J, Terwisscha Van Scheltinga AC, Turner NJ, Dijkstra BW (2016) Structural basis of the substrate range and enantioselectivity of two (*S*)-selective ω-transaminases. Biochemistry 55:4422–4431. 10.1021/acs.biochem.6b00370 27428867 10.1021/acs.biochem.6b00370

[CR56] Vikhrankar SS, Satbhai S, Kulkarni P, Ranbhor R, Ramakrishnan V, Kodgire P (2024) Enzymatic routes for chiral amine synthesis: protein engineering and process optimization. Biologics 18:165–179 38948006 10.2147/BTT.S446712 PMC11214570

[CR57] Wang Y, Feng J, Dong W, Chen X, Yao P, Wu Q, Zhu D (2021) Improving catalytic activity and reversing enantio-specificity of ω-Transaminase by semi-rational engineering en route to chiral bulky β-Amino Esters. ChemCatChem 13:3396–3400. 10.1002/cctc.202100503

[CR58] Weiß MS, Pavlidis IV, Vickers C, Höhne M, Bornscheuer UT (2014) Glycine oxidase based high-throughput solid-phase assay for substrate profiling and directed evolution of (*R*)- and (*S*)-selective amine transaminases. Anal Chem 86:11847–11853. 10.1021/ac503445y 25321325 10.1021/ac503445y

