# Supplementary material for: Functional and structural insights into a thermostable (S)-selective amine transaminase and its improved substrate scope by protein engineering
Source: Appl Microbiol Biotechnol. 2025 Aug 12;109(1):180. doi: 10.1007/s00253-025-13536-9 (PMC12343752; doi:10.1007/s00253-025-13536-9)
Supplement: Supplementary file 1 — (DOCX 916 KB) [file 253_2025_13536_MOESM1_ESM.docx]

**Supplementary Information**

**Applied Microbiology and Biotechnology**

**Functional and structural insights into a thermostable (*S*)-selective amine transaminase and its improved substrate scope by protein engineering**

*Stefania Patti^1,2 #^, Simone A. De Rose^3#^, Michail N. Isupov^3^, Ilaria Magrini Alunno^1^, Sergio Riva^1^, Erica Elisa Ferrandi^1^*, Jennifer A. Littlechild^3^*, Daniela Monti^1^**

^1^ Istituto di Scienze e Tecnologie Chimiche “G. Natta” (SCITEC), CNR, Milano, Italy

^2^ Department of Pharmaceutical Sciences, University of Milan, Milano, Italy

^3^ Henry Wellcome Building for Biocatalysis, Biosciences, Faculty of Health and Life Sciences, University

of Exeter, Exeter, United Kingdom

^#^ These authors contributed equally to this work

*Corresponding authors

Email - Jennifer A. Littlechild, J.A.Littlechild@exeter.ac.uk; Erica Elisa Ferrandi, erica.ferrandi@scitec.cnr.it; Daniela Monti, daniela.monti@scitec.cnr.it

**Index**

| **Site-directed mutagenesis** | p. 2 |
| --- | --- |
| **Table S1.** Primers used in this study | p. 3 |
| **Table S2.** Mutants expression yields | p. 3 |
| **Table S3.** Sbv333-ATA stability to water-miscible co-solvents | p. 4 |
| **Table S4.** Sbv333-ATA stability in biphasic systems (1:1) | p. 4 |
| **Table S5**. Amine donor screening with wild-type Sbv333-ATA and engineered variants. | p. 5 |
| **Table S6.** Detailed crystallization conditions for all the Sbv333-ATA structures | p. 6 |
| **Figure S1.** Chemical structure of the substrate phenylacetylcarbinol (PAC) and the product analogue norephedrine tested in Sbv333-ATA co-crystallization experiments. | p. 6 |
| **Figure S2.** Chemical structure of aromatic (*R*)-amines with increasing side-chain lengths tested as substrates with Sbv333-ATA variants | p. 6 |
| **Figure S3.** Screening of Sbv333-TA mutants designed for reverse enantioselectivity. | p. 7 |
| **Figure S4.** Deamination of selected aromatic (*R*)-amines catalyzed by W89A Sbv333-ATA variant at different pH values. | p. 7 |
| **Figure S5.** Transaminase amino acid structural alignments of the active site area of Sbv333-ATA, *Silicibacter sp.* tm1040 (PDB: 3FCR), *Silicibacter pomeroyi* (PDB: 3HMU) and *Vibrio fluvialis* (PDB: 4E3Q). | p. 8 |
| **Figure S6:** Schematic overview of stabilising interactions of the Sbv333 gabaculine complex. | p. 9 |
| **References** | p. 9 |

**Site-directed mutagenesis**

Sbv333-TA variants carrying up to two-point mutations were created by a Q5® Site-Directed Mutagenesis Kit (New England BioLabs) and a QuikChange II XL site-directed mutagenesis kit (Agilent) according to the manufacturer’s protocols. Mutagenesis primers (Table S1) were designed using the primer design program suggested by the respective manufacturer (<https://international.neb.com/tools-and-resources/video-library/nebasechanger-designing-primers-for-use-with-the-q5-site-directed-mutagenesis-kit>; https://www.agilent.com/store/primerDesignProgram.jsp).

A typical PCR mixture (25 µL) for the Q5® Site-Directed Mutagenesis Kit consisted of 12.5 µL of Q5 Hot Start High-Fidelity 2X Master Mix, 1.25 µL of 10 µM Forward Primer, 1.25 µL of 10 µM Reverse Primer; 1-25 ng of Template DNA and 9 µL of Nuclease-free Water. After initial denaturation for 30 sec at 98 °C, the cycling program was followed for 25 cycles: 10 sec, 98 °C, denaturation; 10-30 sec, primer annealing at the primer specific optimal annealing temperature; and 20-30 sec/kb, 72 °C. elongation. The final extension step was performed for 2 min at 72 °C. After PCR, the amplified material is added directly to a Kinase-Ligase-DpnI (KLD) enzyme mix for rapid, room temperature circularization and template removal. The mixture is composed by: 1 µL of PCR product; 5 µL of 2X KLD Reaction Buffer; 1 µL of 10X KLD enzyme mix and 3 µL of Nuclease-free Water. The mixture was then incubated at room temperature for 5 min, then transformation into NEB 5-alpha Competent *E. coli* cells was performed. Clones from an overnight growth on agar plates supplemented with kanamycin were inoculated in a 5 mL overnight cultivation (LB media, kanamycin complemented). Plasmids were isolated from the overnight culture using GeneJET Plasmid Miniprep Kit (Thermo Scientific™). To verify the presence of the single point mutation plasmids were sequenced by Eurofins (UK).

A typical PCR mixture (50 µL) for the QuikChange II XL site-directed mutagenesis kit consisted of 5 µL of 10x reaction buffer (12.5 µL), 1 µL of a mixture of deoxynucleoside triphosphates, 3 µL of QuickSolution, 1 µL of *PfuTurbo* DNA polymerase (2.5 U/µL), 10 ng of plasmid DNA and the forward and reverse primers (0.5 µM) and ddH_2_O to a final volume of 50 µL. After initial denaturation for 1 min at 95°C, the cycling program was followed for 18 cycles: 50 sec, 95°C, denaturation; 50 sec, primer annealing at the primer specific optimal annealing temperature; and 1 min/kb (4 min), 68°C, elongation. The final elongation step was performed for 7 min at 68°C. After PCR, the reaction mixtures were digested for 1 h at 37°C with *Dpn* I (10 U/µL), followed by transformation into XL10-Gold ultracompetent cells. Clones from an overnight growth on agar plates supplemented with kanamycin were used for inoculation of a 5 mL overnight cultivation (LB media, kanamycin complemented). Plasmids were isolated from the overnight culture using HiSpeed® Plasmid Midi Kit (Qiagen, Hilden, Germania). To verify the presence of the single point mutation plasmids were sequenced by Bio-Fab Research (Rome).

**Table S1.** Primers used in this study

| Position and mutation | Primer Sequence |
| --- | --- |
| F61A (forward) | GGCGGGCCTCgcgGTCGTGCAGG |
| F61A (reverse) | AGTCCGTCCAGGTACCGC |
| W89A (forward) | CTTCCCCATCgcgTCGTACGCCCACC |
| W89A (reverse) | AAGGCCAGGTCCTGTGCC |
| L60A (forward) | ACTGGCGGGCgcgTTCGTCGTGCAGGCC |
| L60A (reverse) | CCGTCCAGGTACCGCCGC |
| W89Y (forward) | CTTCCCCATCtacTCGTACGCCCACCC |
| W89Y (reverse) | AAGGCCAGGTCCTGTGCC |
| F61C (forward) | CTGCACGACgcaGAGGCCCGCCAGTC |
| F61C (reverse) | GACTGGCGGGCCTCtgcGTCGTGCAG |
| F61V (forward) | CCTGCACGACgacGAGGCCCGCCAG |
| F61V (reverse) | CTGGCGGGCCTCgtcGTCGTGCAGG |
| F23V (forward) | CGACATGCGCGTgacGTGCATCCACAGGT |
| F23V (reverse) | ACCTGTGGATGCACgtcACGCGCATGTCG |
| L60V (forward) | GCACGACGAAgacGCCCGCCAGTCC |
| L60V (reverse) | GGACTGGCGGGCgtcTTCGTCGTGC |
| F23W (forward) | GACATGCGCGTccaGTGCATCCACAGGTGGTCGT |
| F23W (reverse) | ACGACCACCTGTGGATGCACtggACGCGCATGTC |
| D421E (forward) | CGCCGCGGTCctcGGCGCGGC |
| D421E (reverse) | GCCGCGCCgagGACCGCGGCG |
| D421W (forward) | GGTCGCCGCGGTCccaGGCGCGGCAGTAG |
| D421W (reverse) | CTACTGCCGCGCCtggGACCGCGGCGACC |
| F61W (forward) | CGGCCTGCACGACccaGAGGCCCGCCAG |
| F61W (reverse) | CTGGCGGGCCTCtggGTCGTGCAGGCCG |

For heterologous protein production, the isolated plasmids were transformed into *E. coli* BL21(DE3) cells containing plasmid pGRO7 (Takara Bio Inc., Kyoto, Japan) coding for co-chaperons GroES and GroEL. Protein expression yields are reported in Table S2.

**Table S2.** Mutants expression yields

| **Mutation** | **Protein expression yield (mg L_culture_^-1^)** |
| --- | --- |
| F23V | 99 |
| L60A | - |
| L60V | 56 |
| F61A | - |
| F61V | 119 |
| F61C | 99 |
| W89A | 54 |
| W89Y | - |
| Y153W | 38 |
| W89Y/ Y153W | 66 |
| W89A/ F23W | - |
| W89A/ F61W | 68 |
| W89A/ D421E | 82 |
| W89A/ D421W | 97 |

- **Sbv333-TA functional characterization**

**Table S3.** Sbv333-ATA stability to water-miscible co-solvents

| **Time (h)** | **Co-solvents** | **U mL^-1^** | **Residual activity (%)** |
| --- | --- | --- | --- |
| 0 | None | 1.34 | 100 |
| 5 |  | 1.38 | 103 |
| 24 |  | 0.73 | 54 |
| 0 | MeOH 5% | 1.11 | 100 |
| 5 |  | 0.95 | 86 |
| 24 |  | 0.62 | 56 |
| 0 | MeOH 10% | 1.11 | 100 |
| 5 |  | 1.23 | 111 |
| 24 |  | 0.73 | 66 |
| 0 | MeOH 20% | 0.98 | 100 |
| 5 |  | 1.08 | 110 |
| 24 |  | 0.63 | 64 |
| 0 | EtOH 5% | 1.29 | 100 |
| 5 |  | 1.29 | 100 |
| 24 |  | 0.51 | 40 |
| 0 | EtOH 10% | 1.24 | 100 |
| 5 |  | 1.27 | 102 |
| 24 |  | 0.57 | 46 |
| 0 | EtOH 20% | 1.05 | 100 |
| 5 |  | 1.37 | 109 |
| 24 |  | 0.56 | 53 |
| 0 | ACN 5% | 1.16 | 100 |
| 5 |  | 1.08 | 93 |
| 24 |  | 1.02 | 88 |
| 0 | ACN 10% | 1.16 | 100 |
| 5 |  | 1.20 | 103 |
| 24 |  | 0.77 | 66 |
| 0 | ACN 20% | 1.14 | 100 |
| 5 |  | 1.28 | 112 |
| 24 |  | 0.76 | 67 |
| 0 | DMSO 5% | 1.20 | 100 |
| 5 |  | 1.31 | 109 |
| 24 |  | 1.10 | 92 |
| 0 | DMSO 10% | 1.28 | 100 |
| 5 |  | 1.33 | 104 |
| 24 |  | 1.06 | 83 |
| 0 | DMSO 20% | 1.30 | 100 |
| 5 |  | 1.06 | 82 |
| 24 |  | 1.00 | 77 |

**Table S4.** Sbv333-ATA stability in biphasic systems (1:1)

| **Time (h)** | **Solvents** | **U mL^-1^** | **Residual activity (%)** |
| --- | --- | --- | --- |
| 0 | None | 1.00 | 100 |
| 5 |  | 0.95 | 95 |
| 24 |  | 0.65 | 65 |
| 5 | PE | 0.95 | 95 |
| 24 |  | 0.51 | 51 |
| 5 | Tol | 1.12 | 112 |
| 24 |  | 0.71 | 71 |
| 5 | EtOAc | 1.19 | 109 |
| 24 |  | 0.68 | 68 |

**Table S5**. Amine donor screening with wild-type Sbv333-ATA and engineered variants.

| **Enzyme** | **Substrate** | **ΔmAbs min^-1^** | **mU mL^-1^** | **mg mL^-1^** | **mU mg^-1^** |
| --- | --- | --- | --- | --- | --- |
| WT | (*S*)-**1** | 12.49 | 375.64 | 11.5 | 32.66 |
|  | (*S*)-**5** | 12.49 | 375.64 |  | 32.66 |
|  | **7** | 1.66 | 49.92 |  | 4.34 |
|  | **10** | 1.92 | 57.62 |  | 5.01 |
|  | **11** | 21.45 | 645.11 |  | 56.10 |
|  | **12** | 15.88 | 477.59 |  | 41.53 |
|  | **15** | 3.40 | 102.17 |  | 8.88 |
|  | (*S*)-**20** | 7.99 | 240.57 |  | 20.92 |
|  | **21** | 7.86 | 236.24 |  | 20.54 |
| F23V | (*S*)-**5** | 0.61 | 18.22 | 16 | 1.14 |
|  | **11** | 1.29 | 38.83 |  | 2.43 |
| L60V | (*S*)-**1** | 0.33 | 10.03 | 7.4 | 1.36 |
|  | **10** | 0.13 | 4.00 |  | 0.54 |
|  | **12** | 0.41 | 12.47 |  | 1.69 |
|  | **19** | 0.13 | 4.14 |  | 0.56 |
| F61C | (*S*)-**1** | 20.46 | 615.34 | 19.9 | 30.92 |
|  | (*S*)-**3** | 1.25 | 37.71 |  | 1.90 |
|  | (*S*)-**5** | 22.98 | 691.13 |  | 34.73 |
|  | **7** | 3.47 | 104.39 |  | 5.25 |
|  | **10** | 2.47 | 74.17 |  | 3.73 |
|  | **11** | 10.83 | 325.71 |  | 16.37 |
|  | **12** | 23.16 | 696.54 |  | 35.00 |
|  | **15** | 6.97 | 209.65 |  | 10.54 |
|  | (*S*)-**20** | 7.81 | 234.86 |  | 11.80 |
|  | **21** | 6.46 | 194.20 |  | 9.76 |
| W89A | (*S*)-**1** | 3.35 | 100.78 | 12 | 8.40 |
|  | (*S*)-**3** | 2.17 | 65.32 |  | 5.44 |
|  | **4** | 7.31 | 219.76 |  | 18.31 |
|  | (*S*)-**5** | 4.54 | 136.51 |  | 11.38 |
|  | **7** | 2.80 | 84.06 |  | 7.01 |
|  | **10** | 0.19 | 5.69 |  | 0.47 |
|  | **11** | 17.32 | 520.90 |  | 43.41 |
|  | **12** | 15.91 | 478.50 |  | 39.87 |
|  | **15** | 2.77 | 83.16 |  | 6.93 |
|  | **21** | 2.99 | 90.20 |  | 7.52 |
| W89A/L60V | (*S*)-**1** | 0.22 | 6.70 | 9.52 | 0.70 |
|  | (*S*)-**3** | 0.51 | 15.32 |  | 1.61 |
|  | (*S*)-**5** | 0.34 | 10.27 |  | 1.08 |
|  | **7** | 0.14 | 4.19 |  | 0.44 |
|  | **10** | 0.29 | 8.82 |  | 0.93 |
|  | **11** | 1.20 | 36.21 |  | 3.80 |
|  | **19** | 0.17 | 5.18 |  | 0.54 |

**Table S6.** Detailed crystallization conditions for all the Sbv333-ATA structures

| **Sbv333-ATA** | **Crystallization conditions** |
| --- | --- |
| Native | 0.2 M potassium thiocyanate; 0.1 M Bis Tris propane 6.5; 20 % w/v PEG 3350 |
| Gabaculine | 10 mM Gabaculine 0.2 M potassium thiocyanate 0.1 M Bis Tris propane 6.5 20 % w/v PEG 3350 |
| Phenylacetylcarbinol (PAC) | 10 mM PAC; 0.1 Imidazole; MES monohydrate (acid) pH 6.5; 30% Precipitant mix (40% v/v (PEG 500* MME; 20 % w/v PEG 20000); Additives: 0.2M D-Glucose; 0.2M D-Mannose; 0.2M DGalactose; 0.2M L-Fucose; 0.2M D-Xylose; 0.2M N-Acetyl-D-Glucosamine |
| Norephedrine | 10 mM 1s2s Pseudoephedrine; 0.1 M Bis-Tris propane, pH 6.5; 20 % w/v PEG 3350; 0.2 M Sodium iodide |
| W89A | 0.3M Diethylene glycol; 0.3M Triethylene glycol; 0.3M Tetraethylene glycol; 0.3M Pentaethylene glycol; Tris (base) BICINE pH 8.5; 30% Precipitant mix (40% v/v PEG 500* MME; 20% w/v PEG 20000) |
| F61C | 0.2 M Magnesium chloride hexahydrate; 0.1 M Tris 7.5; 25 % w/v PEG 2000 MME |

**Figure S1.** Chemical structure of the substrate phenylacetylcarbinol (PAC) and the product analogue norephedrine tested in Sbv333-ATA co-crystallization experiments.

**Figure S2.** Chemical structure of aromatic (*R*)-amines with increasing side-chain lengths tested as substrates with Sbv333-ATA variants.

**Figure S3.** Screening of Sbv333-TA mutants designed for reverse enantioselectivity.


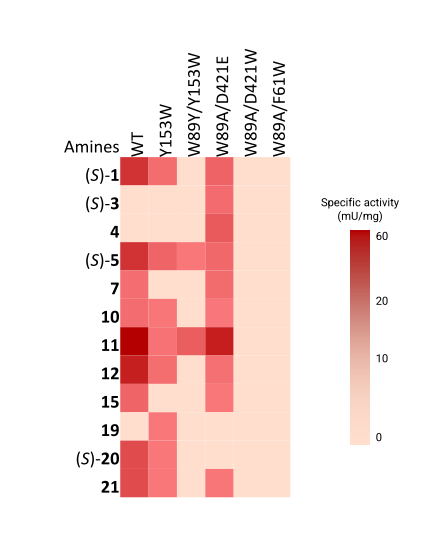


**Figure S4.** Deamination of selected aromatic (*R*)-amines catalyzed by W89A Sbv333-ATA variant at different pH values.


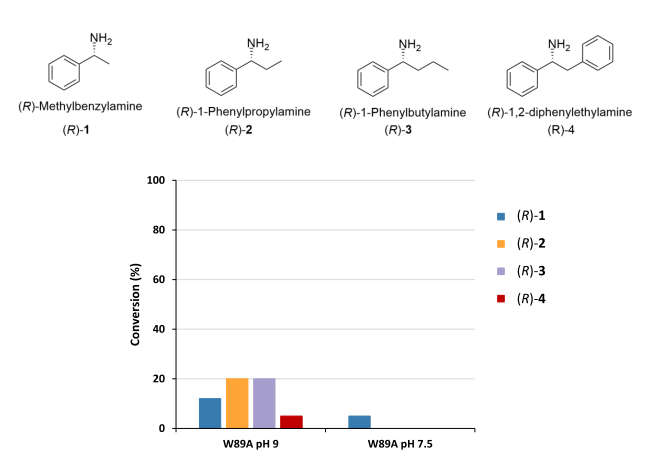


**Figure S5.** Transaminase amino acid structural alignments of the active site area of Sbv333-ATA, *Silicibacter sp.* tm1040 (PDB: 3FCR), *Silicibacter pomeroyi* (PDB: 3HMU) and *Vibrio fluvialis* (PDB: 4E3Q). The proteins Sbv333-ATA, 3FCR, 3HMU and 4E3Q are shown as cyan, green, yellow and purple sticks, respectively. The oxygen and nitrogen atoms of different residues are coloured red and blue, PLP is shown as a grey ball and stick model.


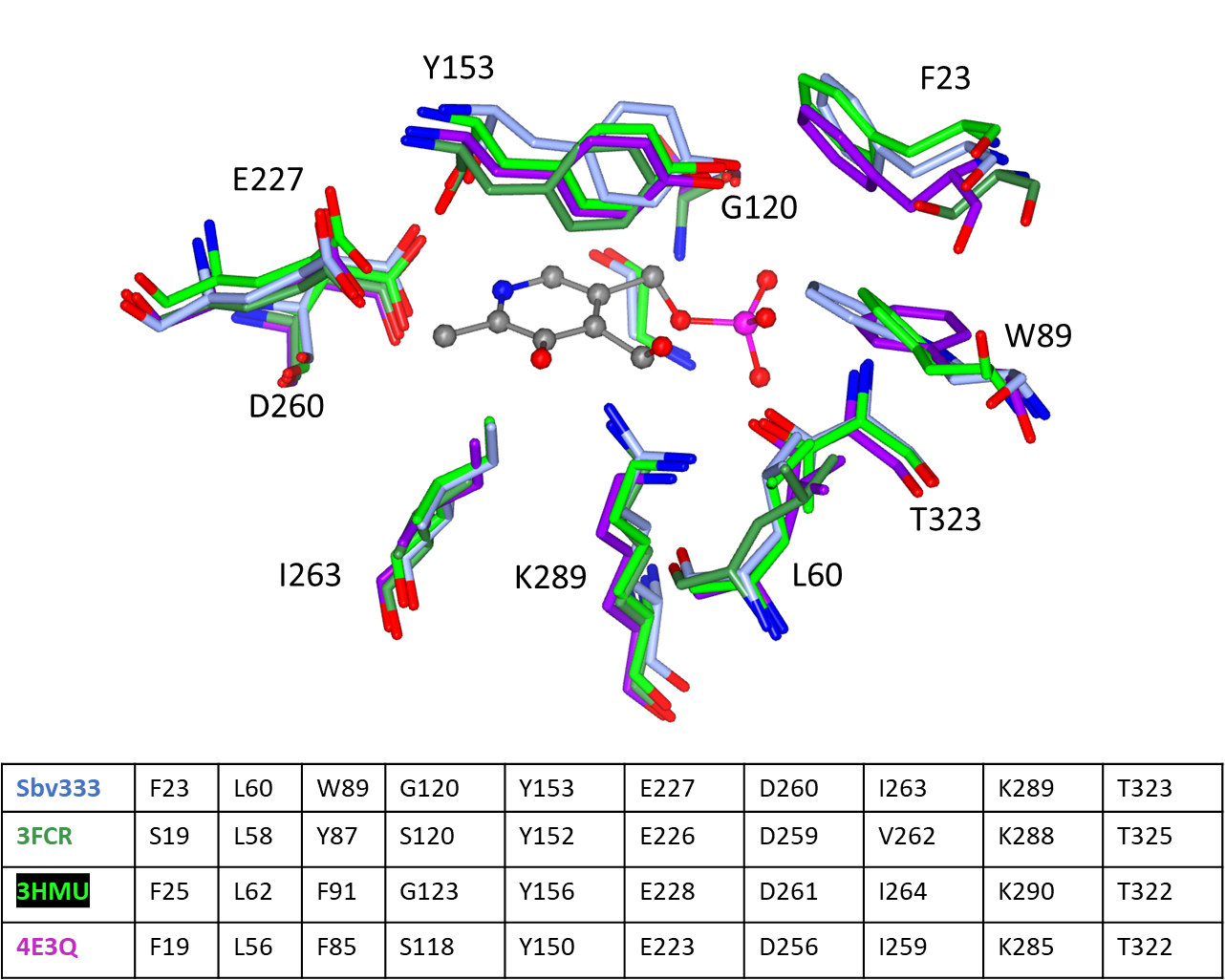


**Figure S6:** Schematic overview of stabilising interactions of the Sbv333 gabaculine complex. (**A**) a portion of the large pocket showing the residue range from L166 to L176. (**B**) The small pocket with all the residues and the gabaculine molecule shown. Hydrogen bonds are shown as green dashes and their distances indicated. Residues involved in hydrophobic interaction are shown as red radiated semicircle. Figure generated using LigPlot+ (Wallace et al. 1995; Laskowski and Swindells 2011).


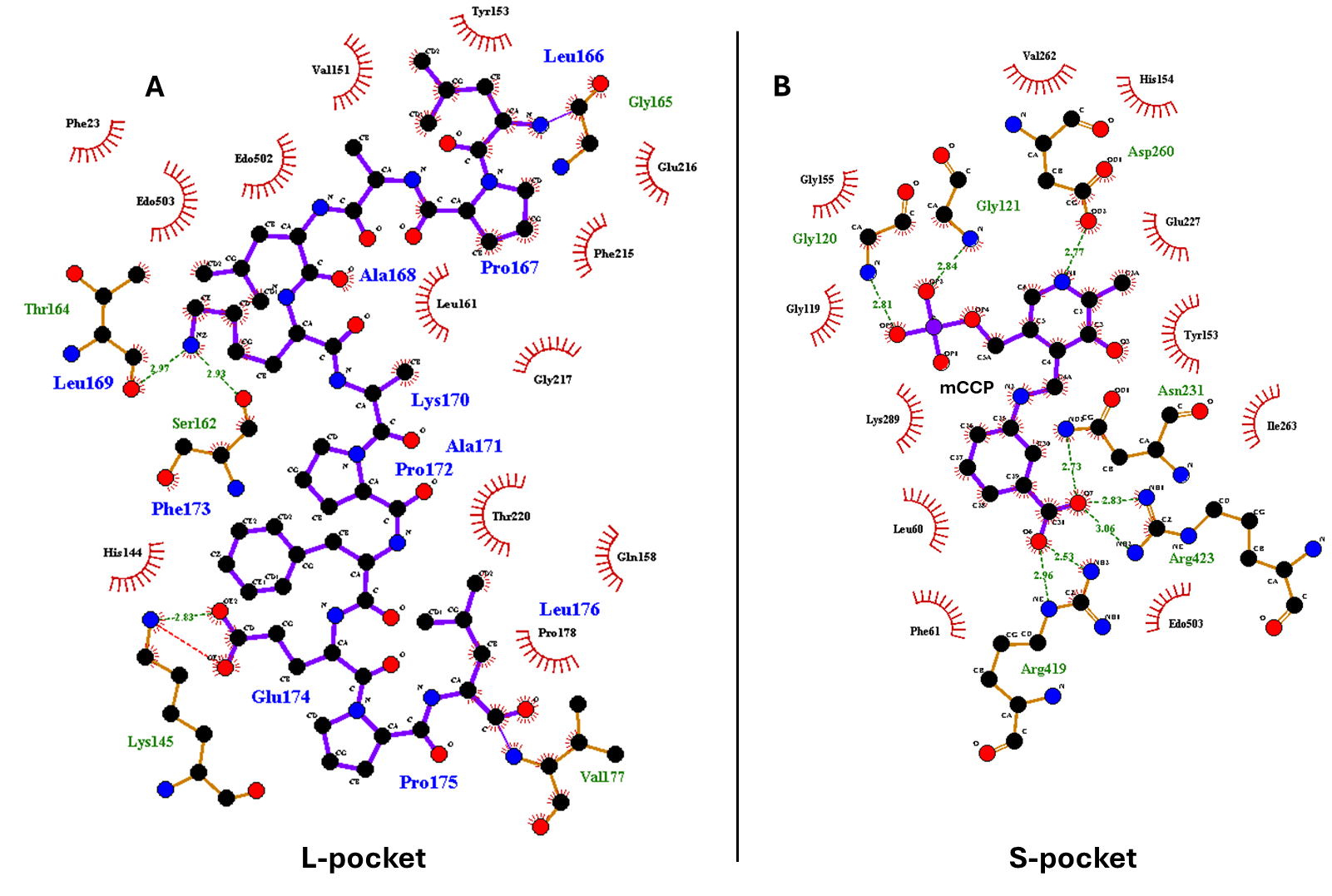


**References**

Laskowski RA, Swindells MB (2011) LigPlot+: Multiple Ligand–Protein Interaction Diagrams for Drug Discovery. J Chem Inf Model 51:2778–2786. https://doi.org/10.1021/ci200227u

Wallace AC, Laskowski RA, Thornton JM (1995) LIGPLOT: a program to generate schematic diagrams of protein-ligand interactions. Protein Eng Des Sel 8:127–134. https://doi.org/10.1093/protein/8.2.127
